# The Relationship between Anti-merozoite Antibodies and Incidence of *Plasmodium falciparum* Malaria: A Systematic Review and Meta-analysis

**DOI:** 10.1371/journal.pmed.1000218

**Published:** 2010-01-19

**Authors:** Freya J. I. Fowkes, Jack S. Richards, Julie A. Simpson, James G. Beeson

**Affiliations:** 1 Division of Infection and Immunity, The Walter and Eliza Hall Institute of Medical Research, Parkville, Victoria, Australia; 2 Department of Medical Biology, University of Melbourne, Victoria, Australia; 3 Centre for Molecular, Environmental, Genetic and Analytic Epidemiology, University of Melbourne, Victoria, Australia; University of Oxford, United Kingdom

## Abstract

**Background:**

One of the criteria to objectively prioritize merozoite antigens for malaria vaccine development is the demonstration that naturally acquired antibodies are associated with protection from malaria. However, published evidence of the protective effect of these antibodies is conflicting.

**Methods and Findings:**

We performed a systematic review with meta-analysis of prospective cohort studies examining the association between anti-merozoite immunoglobin (Ig) G responses and incidence of *Plasmodium falciparum* malaria. Two independent researchers searched six databases and identified 33 studies that met predefined inclusion and quality criteria, including a rigorous definition of symptomatic malaria. We found that only five studies were performed outside sub-Saharan Africa and that there was a deficiency in studies investigating antibodies to leading vaccine candidates merozoite surface protein (MSP)-1_42_ and erythrocyte binding antigen (EBA)-175. Meta-analyses of most-studied antigens were conducted to obtain summary estimates of the association between antibodies and incidence of *P. falciparum* malaria. The largest effect was observed with IgG to MSP-3 C terminus and MSP-1_19_ (responders versus nonresponders, 54%, 95% confidence interval [CI] [33%–68%] and 18% [4%–30%] relative reduction in risk, respectively) and there was evidence of a dose-response relationship. A tendency towards protective risk ratios (RR<1) was also observed for individual study estimates for apical membrane antigen (AMA)-1 and glutamate-rich protein (GLURP)-R0. Pooled estimates showed limited evidence of a protective effect for antibodies to MSP-1 N-terminal regions or MSP-1-EGF (epidermal growth factor-like modules). There was no significant evidence for the protective effect for MSP-2 (responders versus nonresponders pooled RR, MSP-2_FC27_ 0.82, 95% CI 0.62–1.08, *p* = 0.16 and MSP-2_3D7_ 0.92, 95% CI 0.75–1.13, *p* = 0.43). Heterogeneity, in terms of clinical and methodological diversity between studies, was an important issue in the meta-analysis of IgG responses to merozoite antigens.

**Conclusions:**

These findings are valuable for advancing vaccine development by providing evidence supporting merozoite antigens as targets of protective immunity in humans, and to help identify antigens that confer protection from malaria. Further prospective cohort studies that include a larger number of lead antigens and populations outside Africa are greatly needed to ensure generalizability of results. The reporting of results needs to be standardized to maximize comparability of studies. We therefore propose a set of guidelines to facilitate the uniform reporting of malaria immuno-epidemiology observational studies.

*Please see later in the article for the Editors' Summary*

## Abstract 2

A systematic review and meta-analysis examining the association between anti-merozoite antibody responses and incidence of *Plasmodium falciparum* malaria by Freya Fowkes and colleagues aids identification of antigens that confer protection from malaria.

## Editors' Summary

**Background:**

*Plasmodium falciparum* malaria, a mosquito-borne parasitic infection, kills about one million people every year. Around a week after an infected mosquito has bitten a person, “merozoites” (one of the life-stages of the parasite) infect the person's red blood cells where they replicate and then burst out and infect more red blood cells. Rapid replication of parasites can occur in the bloodstream, leading to massive numbers of parasites that can damage vital organs. Although individuals can lower their risk of becoming infected with malaria parasites by avoiding mosquito bites, a vaccine is urgently needed to reduce the global burden of malaria. When malaria parasites infect a person for the first time, the human immune system begins to produce antibodies, proteins that recognize molecules (antigens) on the parasite's surface and that act directly or cooperate with other parts of the immune system to kill malaria parasites. The production of these “naturally acquired” antibodies is initially slow so the individual can become ill when infected. However, because the immune system “remembers” how to make the antibodies, its response to subsequent infections is quicker. The levels of these antibodies also build up with each infection and become more effective at killing parasites. Vaccines, which contain malaria antigens, “prime” the immune system to respond rapidly to malaria infections and produce high concentrations of antibodies to prevent the infection from causing serious illness.

**Why Was This Study Done?:**

A malaria vaccine that stimulates an efficient immune response against merozoites would limit the severity of malarial infections and prevent many deaths but no one knows which (if any) of the antigens on merozoites stimulate a protective immune response. Although many different types of antibodies are produced by the immune system, only some of these are effective in protecting against malaria. By investigating whether there is an association between naturally acquired antibodies, which recognize specific candidate antigens, and protection from malaria in populations living in areas where malaria is endemic (always present), vaccine developers can get an idea about which antigens to include in their vaccines. Although many of these “malaria immuno-epidemiological studies” have been undertaken, their results are somewhat conflicting. In this study, the researchers reanalyze these results by doing a systematic review (a study that uses predefined criteria to identify all the research on a specific topic) and a meta-analysis (a statistical method for combining the results of several studies). The researchers evaluated studies of the relationship between anti-merozoite antibodies and the incidence (the number of new cases of a disease in a population per year) of *P. falciparum* malaria in naturally exposed populations in different regions of the world.

**What Did the Researchers Do and Find?:**

The researchers' search of the published literature yielded 33 studies in which the incidence of malaria had been recorded over time in groups of people in whom levels of antibodies to specific merozoite antigens had been measured. These studies measured antibodies at the start of the study and examined the subsequent risk of malaria over several months of follow-up (these are known as prospective cohort studies). All but five of the studies were performed in Africa, and very few merozoite antigens had been well-studied in different populations, or studied at all. Of note, very few studies had examined naturally acquired antibodies to some leading vaccine candidates (for example, only one study considered antibodies to MSP-1_42_, a leading vaccine candidate). Conversely, the association between malaria incidence and antibodies to the antigen MSP-1_19_, which has been included in only one candidate vaccine, was frequently studied. In their meta-analyses, the researchers found that among people with antibodies to the merozoite antigens MSP-3 (C-terminal region) and MSP-1_19_, the risk of developing *P. falciparum* malaria was reduced by 54% and 18%, respectively, compared to people without antibodies to these antigens. There was also some evidence of a reduced risk of malaria for people with antibodies to AMA1 and GLURP. For other merozoite antigens, MSP1 (N-terminal region) and MSP2, there was either weak or no evidence for a protective effect of naturally acquired antibodies.

**What Do These Findings Mean?:**

These findings suggest that merozoite antigens are important targets of protective immunity in people who are naturally exposed to malaria and also suggest which of these antigens might be included in vaccines. However, the findings are limited by the small number of studies identified by the researchers and additional prospective cohort studies are clearly needed to guide vaccine development. These studies will need to include a larger number of lead antigens and populations outside Africa to ensure their generalizability, note the researchers. Furthermore, efforts will need to be made to ensure greater consistency between studies to improve the ability to compare results between different studies, which was a challenge in performing this study. To this end, the researchers propose a set of guidelines that, if followed, should make it easier to compare the results of different malaria immune-epidemiology studies in the future and thus lead to better identification of candidate vaccine antigens.

**Additional Information:**

Please access these Web sites via the online version of this summary at http://dx.doi.org/10.1371/journal.pmed.1000218.

- Information is available from the World Health Organization on malaria (in several languages) and on the development of malaria vaccines
- The US Centers for Disease Control and Prevention provides information on malaria (in English and Spanish)
- Information is available from the Wellcome Trust on all aspects of malaria, including vaccine development
- The Malaria Vaccine Initiative provides information on the development of malaria vaccines and on ongoing trials
- MedlinePlus provides links to additional information on malaria (in English and Spanish)

## Introduction

Malaria caused by *Plasmodium falciparum* is a leading cause of mortality and morbidity globally, particularly among young children. After repeated exposure, individuals develop effective immunity that controls blood-stage parasitaemia, thereby reducing clinical symptoms and life-threatening complications (reviewed in [1]). Antibodies are important mediators of acquired immunity to malaria as evidenced by experimental animal models and, most importantly, passive transfer studies in which antibodies from malaria-immune adults were successfully used to treat patients with severe malaria [2],[3]. Antibodies to merozoite antigens are considered important targets of protective antibodies and are thought to function in vivo by inhibiting merozoite invasion of erythrocytes, opsonizing merozoites for phagocytosis, and antibody-dependent cellular inhibition [4]–[7]. However, it is unclear which merozoite antigens are important targets of naturally acquired immunity.

A number of merozoite antigens have established roles in erythrocyte invasion and some have been identified as targets of human invasion-inhibition antibodies or antibody-dependent cellular inhibition in vitro [8]–[15]. Merozoite surface proteins (MSPs) are thought to be involved in the initial attachment of the merozoite to the erythrocyte surface (e.g., MSP-1) and apical membrane antigen-1 (AMA-1) has been implicated in apical reorientation of the merozoite prior to invasion. Two invasion ligand families present in the apical organelles, the erythrocyte binding antigens (e.g., EBA175, EBA181, EBA140) and *P. falciparum* reticulocyte-binding homologues are also required for invasion [16]. There are numerous surface proteins with no known function including MSP-2, MSP-3, MSP-4, and glutamate-rich protein (GLURP) [16]. Genetic polymorphisms exist in most antigens and some can be grouped into major allelic types. Many of these antigens are currently being evaluated or developed as candidates for inclusion in an erythrocytic-stage malaria vaccine [17].

There are several criteria that can be used to objectively prioritize known and predicted antigens for vaccine development [17]. These include the demonstration that antibodies against these antigens inhibit *P. falciparum* growth in vitro, or are protective in animal models, and the demonstration that naturally acquired antibodies are associated with protection from symptomatic disease in malaria endemic populations. Consequently, numerous epidemiological studies have investigated the role of merozoite surface antigens as targets of human immunity. However, the epidemiological evidence of the protective effect of naturally acquired anti-merozoite responses is conflicting. There are numerous potential reasons for the inconsistencies in the estimates of protection. In malaria endemic areas the rate at which natural immunity develops is dependent on the intensity and stability of exposure to *P. falciparum*, with immunity to severe and mild disease developing more rapidly in areas with higher transmission [1],[18]. Differences in the acquisition of immunity may influence associations between specific responses and immunity. Furthermore, the prevalence of the major allelic types of specific antigens and subsequent acquisition of allele-specific immunity may be different across populations. The alleles represented in recombinant proteins used for determining antibody responses varies between studies in addition to the preparation of antigens used in immunoassays. Most importantly, the study designs used to investigate the associations between antibody responses and *P. falciparum* malaria studies vary considerably among the published literature. Evidence quoted in the literature regarding the protective role of antigen-specific antibodies is often based on data from cross-sectional or case-control studies. Examining the association of antibody responses with parasitological and clinical outcomes determined at a single time point, or in individuals who have already developed disease, makes the establishment of causality problematic. The highest level of evidence of causality in observational studies comes from prospective cohort studies in which a temporal relationship can be established between exposure and outcome.

We performed a systematic review, with meta-analyses, of cohort studies to determine the association of antibody responses to merozoite surface antigens with incidence of *P. falciparum* malaria in naturally exposed populations, and to identify factors that may account for differences in reported findings. The broad aim of this study was to advance our understanding of naturally acquired immunity to malaria and to contribute to rational vaccine development.

## Materials and Methods

We performed a systematic review of the published literature according to the Meta-analysis Of Observational Studies in Epidemiology (MOOSE) guidelines for the conduct of meta-analyses of observational studies [19]. Results are reported according to the recently published PRISMA guidelines (Preferred Reporting Items for Systematic Reviews and Meta-Analyses; http://www.prisma-statement.org; Text S1). The study protocol was developed by FJIF, JAS, and JGB.

### Search Methods for Identification of Studies

PubMed, Web of Science, Scopus, Google Scholar, African Index Medicus, and LILACS (Latin American and Caribbean Health Sciences Literature) (all years, ending 31 January 2009) were searched for studies examining the association of antibody responses to merozoite antigens with *P. falciparum* malaria. Key words included: MSP, merozoite surface protein, MSA, merozoite surface antigen, GLURP, glutamate-rich protein, serine repeat antigen, SERA, S-Antigen, ABRA, AMA, apical membrane antigen, EBA, erythrocyte binding antigen, rhoptry, malaria, *P. falciparum*, immunity, antibodies, IgG, cohort, longitudinal, incidence, risk, epidemiology, vaccine. The key words variant surface antigen (VSA) were also used because merozoite antigens are sometimes used as comparative antigens in studies investigating VSAs. The reference lists of obtained papers were searched for further studies. Studies reported in languages other than English were included.

### Criteria for Considering Studies for This Review

#### Study designs

The criteria for inclusion of studies were population-based prospective studies and population-based treatment to reinfection studies. Population-based cross-sectional studies to determine prevalence were excluded because causality cannot be established. Case-control studies, hospital-based studies, and vaccine efficacy trials of blood-stage vaccines were also excluded because of the rigorous inclusion and exclusion criteria applied during these studies, such that the participants would not be representative of the general population.

#### Study participants

The criterion for inclusion of participants was individuals living in malaria endemic areas. Studies restricted to pregnant women and/or children <1 y (including maternal transfer studies) were excluded to remove the confounding effect of maternal transferred immunity. Studies where individuals were selected according to their *P. falciparum* status and studies investigating returned travellers or transmigrants were also excluded as they would not be representative of the general population.

#### Antibody measures

Total immunoglobulin G (IgG) responses to recombinant or synthetic defined merozoite antigens measured at baseline (i.e., time 0) were considered. IgG responses to full length proteins, processing products, and defined regions of merozoite antigens were included, but IgG responses to peptides that represent undefined regions or incomplete domains or subdomains of antigens were excluded.

#### Malaria outcome measures

The following malaria outcome measures during follow-up were included: high density *P. falciparum* infection (≥5,000/µl), symptomatic *P. falciparum* malaria, severe *P. falciparum* malaria, and *P. falciparum* malaria-associated mortality. In treatment-to-reinfection studies *P. falciparum* reinfection was also included as an outcome. Newly established blood-stage infection must have been differentiated from treatment failure by either PCR or documented clearance of infection within a specified time frame appropriate for the chosen antimalarial.

#### Quality criteria

The minimum quality criteria for inclusion in the review were that: detection of malaria was by active case detection (ACD) and/or passive case detection (PCD); parasitaemia was confirmed by slide microscopy, rapid detection kit, or PCR; symptomatic malaria was defined as fever and/or history of fever (within the past 72 h) plus a high density parasitaemia threshold (to increase specificity because low-grade parasitaemia is common in most settings); severe malaria was defined by the World Health Organization criteria and other causes of morbidity excluded; and other common causes of mortality excluded before a diagnosis of malaria-associated mortality [20],[21].

### Selection of Studies

Review authors (FJIF, JSR, and JGB) identified possible studies, FJIF and JSR assessed the methodological quality of included studies independently, with discrepancies resolved by discussion with JGB.

#### Effort to include all available studies and data

Authors of studies that had defined a case of symptomatic malaria as fever and/or history of fever plus a *P. falciparum* parasitaemia of any density (i.e., did not meet quality criteria of fever plus a high density threshold) were invited to provide estimates or data meeting the quality criteria. Some studies had analysed antibody levels at baseline as the outcome variable, comparing baseline levels in those who had or did not have *P. falciparum* malaria during follow-up. For these studies, data was extracted and reanalysed so that malaria was the outcome variable and related to antibodies at baseline. If the raw data were not presented, authors of the study were invited to reanalyse or provide data for the inclusion of their study in the systematic review. In addition, we contacted several authors whose studies did not meet the inclusion criteria yet contained data that were eligible for the systematic review. These were the authors of studies that had measured antibody responses after baseline (i.e., examined the association of antibody responses with malaria cases diagnosed both retrospectively and prospectively) and invited them to provide estimates or data concerning prospective *P. falciparum* incidence only. We also contacted authors who had restricted analysis to individuals who were parasite positive at baseline and invited them to provide estimates or data on the whole cohort where possible. If authors were unable to provide estimates or data, the study was classified as not meeting inclusion and/or quality criteria and excluded from the systematic review.

### Data Analysis

#### Data collection

Measures of association (odds ratios [ORs], risk ratios [RRs], incidence rate ratios [IRRs], or hazard ratios [HRs]) and their 95% confidence intervals (CIs) were extracted or derived using data reported in the publications. Data extraction was performed independently by FJIF and JSR, using proforma designed by FJIF, JAS, and JGB. The investigators of the original studies were contacted if relevant information on eligibility or key study data were not available in the published report. An email was sent to authors explaining the nature of the systematic review and the information required together with proforma. If the author did not respond within three email attempts then no further action was taken. Where a study does not provide measures of association (or they could not be calculated with the information provided), the study results will be described only in narrative terms.

#### Standardization of antibody measures

A major issue in reviewing the published results of different epidemiologic studies examining the relation between an exposure variable and risk of the outcome is that the results are presented in many different ways. Determining antibody levels by enzyme-linked immunosorbent assay (ELISA) does not produce a common metric measurement among studies. Individuals can be classified as “responders” or “nonresponders” relative to a negative control (unexposed sera) within each study. Study-specific comparisons of these exposure variables can then be pooled. However, categories based on arbitrary cut-offs (including categories of responders based on statistical rankings) cannot be pooled across studies.

For studies where the antibody measures were analysed as continuous exposure variables we either asked the authors to reanalyse their data by collapsing the antibody data into categories or asked them to provide the standard deviation of the data so we could calibrate the estimate to represent the relative change in the risk of malaria associated with a change of one standard deviation of the antibody level. For log transformed antibody data we used log base 2 so that the relative change in malaria risk corresponds to a doubling of antibody level.

#### Standardization of malaria outcome measures

ORs considerably overestimate the RR, if the incidence risk is >20%, which is often the case in highly malaria endemic areas [22]. Thus, RR, HR, and IRR were extracted or calculated where possible, or unadjusted ORs were converted to RR using the method of Zhang and Yu [23]. RR, HR, and IRR are hereinafter denoted as RR. A RR equal to 1 occurs when the incidence risk of malaria is equal for those with antibody responses (responders) and those without (nonresponders), and when the incidence risk is unchanged for 2-fold increases in the antibody levels. Where possible, estimates adjusted for demographic variables, spatial confounders, *P. falciparum* parasitaemia (at baseline or preseason), and/or bed net use are reported. Estimates adjusted for other anti-merozoite antibodies (including antibodies to schizont protein extract) are not reported because antibody responses are typically highly correlated making it difficult to estimate their individual regression coefficients reliably; in these cases unadjusted estimates are reported. For all malaria outcomes the study-assigned *P. falciparum* definitions were used.

Our aim was to obtain a single RR estimate for each study. If antibody responses to the same antigen, in the same population-based study, were reported in several publications, results from the largest sample size were used. Separate estimates were obtained for the RR associated with AMA-1 (full-length ectodomains of FVO [pro-DI-DII-DIII] and 3D7 [DI-DII-DIII]), EBA-175 (all regions including F1 and F2), GLURP (R0, R1, and R2 fragments), MSP-2 (full length 3D7, full length FC27, and C terminus), MSP-3 (full length 3D7, full length K1, and the conserved C terminus). For MSP-1, separate analyses were done for each region and allelic type (MSP-1-block 1 [MAD20], MSP-1-block 2 [K1-like (3D7), MAD20-like [MAD20], and RO33-like [RO33]), and processing fragments (MSP-1_42_, MSP-1_19_ [including MSP-1-EGF1, MSP-1-EGF2]). Estimates from the above-mentioned regions/alleles were used to ensure maximum comparability between studies. Separate analyses were not done for MSP-3-Ct or MSP-1_19_ alleles because of the conserved nature of MSP-3-Ct and MSP-1_19_ (similarly EGF domains). For these antigens, if responses to multiple alleles were investigated in the same study, the most common circulating allele in the population was included in the meta-analysis.

#### Meta-analysis

Where there were sufficient data, a pooled summary statistic for each malaria outcome was calculated using either a fixed-effect or random-effects model. The standard error of the natural logarithm (ln) of the RR was calculated using the formula (ln[upper limit of CI]−ln[RR])/1.96. Heterogeneity between studies was tested with the *I* ^2^ statistic [24]. If the *I* ^2^ statistic was ≤30%, a meta-analysis based on a fixed-effect model was conducted; otherwise the random-effects model was used. When the *I* ^2^ statistic was >75% and/or the lower 95% confidence limit was between 50%–100%, the studies were not combined [25]. When statistical heterogeneity was noted it was evaluated by fitting meta-regression models to the log-transformed individual study RRs.

Clinical and methodological heterogeneity was explored using prespecified variables to minimize spurious findings. Variables evaluated included study design (prospective cohort, treatment-to-reinfection), length of follow-up, age of study participants (dichotomous variable: adults and children, children only), malaria endemicity (perennial, seasonal, perennial with seasonal peaks), source of malaria cases (dichotomous variable: ACD only, PCD, and ACD), definition of symptomatic malaria, preparation of antigen (allele, expression vector, tag), and method of antibody determination (ELISA, microarray). Influence analysis was also performed whereby pooled estimates were calculated omitting one study at a time. Where possible, publication bias was assessed visually by plotting a funnel plot [26]; publication bias is unlikely if the funnel plots shows a symmetrical inverted V shape [27]. All analyses were performed using the open source statistical package, R 2.9.0 (R Foundation for Statistical Computing).

## Results

### Identification and Description of Included Studies

**Figure 1:**
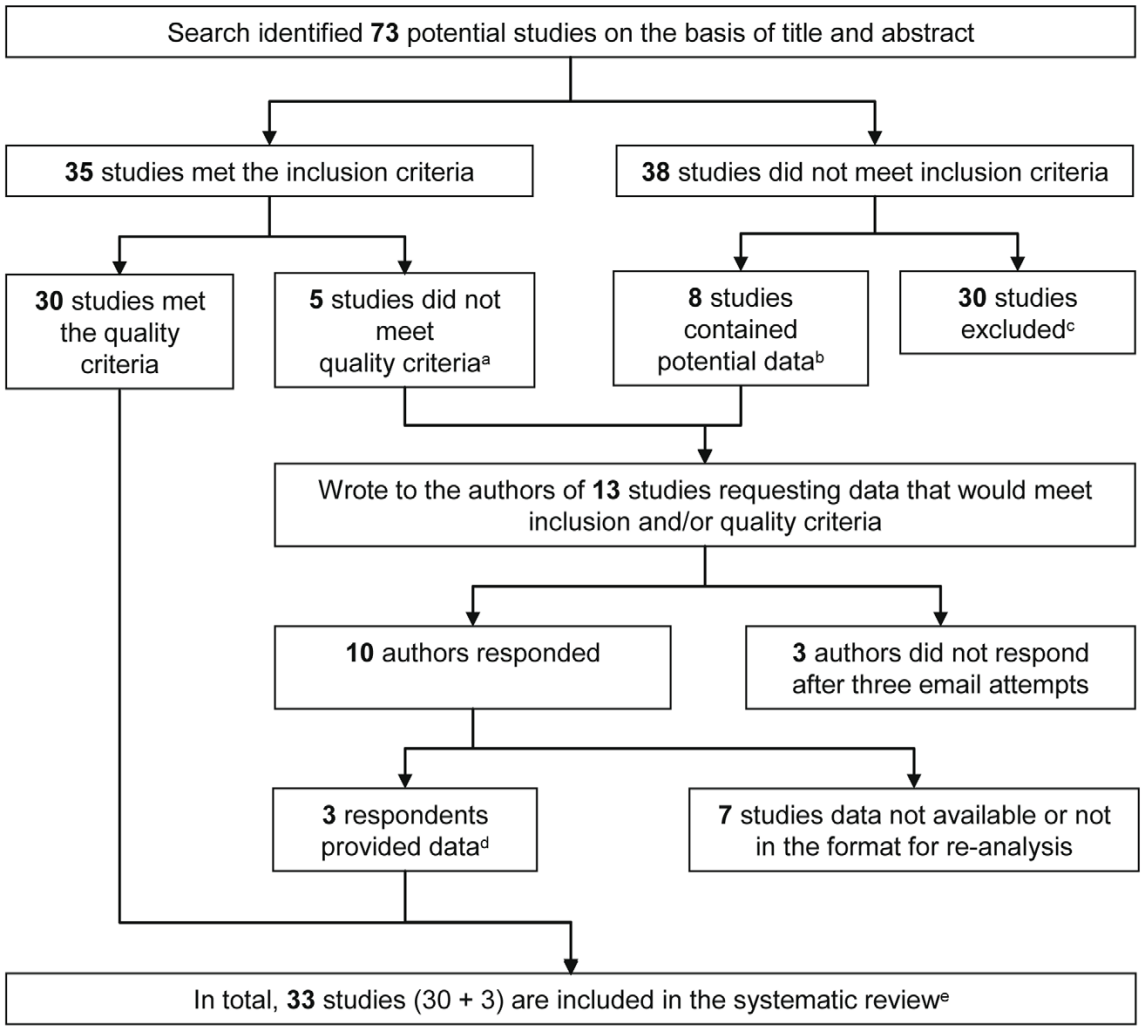
Flow chart of study identification. Details of excluded studies can be found in Text S2. ^a^Definition of symptomatic malaria did not meet protocol definition; ^b^Analysed retro- and prospectively collected clinical data (*n* = 3), analysed antibody levels as outcome (*n* = 4,) and data presented on *P. falciparum* positive individuals only (*n* = 1); ^c^Reasons for exclusion: Data from seroprevalence surveys (*n* = 15); hospital-based study/recruited cases based on clinical/parasitemic status (*n* = 6); did not include malaria outcome of interest (*n* = 5); mother/infant studies (*n* = 3); measured IgG responses to undefined regions of antigens (*n* = 1); ^d^Scopel et al. (2007) provided data using a definition of symptomatic malaria that met our quality criteria, Sarr et al. (2006) provided data so *P. falciparum* could be analysed as outcome, and Osier et al. (2008) provided estimates for the whole cohort, whereas the manuscript originally presented data from *P. falciparum*-positive individuals only [58]–; ^e^The characteristics of included studies are given in Table 1.

Figure 1 outlines identification of studies for this systematic review. The literature search identified 73 potentially relevant studies, of which only 30 fulfilled the inclusion and quality criteria (details of excluded studies can be found in Text S2) [28]–[57]. We obtained further data from three studies after contacting authors (Figure 1), giving a total of 33 studies to be included in the systematic review [58]–[60].

The 33 studies reported data obtained from 14 separate prospective cohort studies and six separate treatment-to-reinfection population studies (Tables 1 and 2, respectively) indicating that multiple publications arise from a single population-based study. For the purpose of this review we shall refer to each publication as a study. The majority of studies report data from Africa (28/33; 84.8%), with three in Papua New Guinea, one in Asia, and one from South America. Study size ranged from 80 to 1,071 participants (median = 280) and duration of participant follow-up ranged from 3 to 18 mo (median = 6). The association of antibody responses to MSP-1 (including processing fragments and defined blocks), MSP-2, and MSP-3, AMA-1, EBA-175, and GLURP with incidence risk of *P. falciparum* malaria was examined in 19, eight, seven, five, three, and six studies, respectively. Details of recombinant proteins and sero-prevalences can be found in Text S2 (Tables A and B). All studies measured total IgG by ELISA with the exception of Gray et al. (2007), who measured IgG by microarray [40]. Symptomatic *P. falciparum* malaria during follow-up was the most common outcome, examined in 29 studies; with reinfection and high density infection during follow-up examined in five and three studies, respectively. No study examined the association of anti-merozoite responses with incidence risk of severe *P. falciparum* malaria or *P. falciparum* malaria-associated mortality.

**Table 1:** Characteristics of prospective studies included in the systematic review by country.

Country	Study: Author, Year [Reference]	Province	Follow-up (mo)	Population		Merozoite IgG Response	*P. falciparum* Outcome	
				Sample Size	Age (y)		Source	Incidence Outcome (Cumulative Incidence %)
**Brazil**	Scopel, 2007 [58]	Acre	15	356	5–65	MSP-2	ACD, PCD	Symptomatic *Pf* c (6.5)
**Burkina Faso**	Meraldi, 2004 [28]	Kadiogo	7	293	0.5–9	GLURP, MSP-3	ACD	Symptomatic *Pf* d (49)
	Nebie, 2008 [29]	Bazega	4	286	0.5–15	AMA-1, GLURP, MSP-1_19_, MSP-3	ACD	Symptomatic *Pf* d (41)
	Nebie, 2008 [30]	Bazega	4	360	0.5–10	GLURP, MSP-3	ACD	Symptomatic *Pf* d (DNS)
**The Gambia**	Conway, 2000 [31] a	Upper River	5	337	3–7	MSP-1_19_, MSP-1-BL1, MSP-1-BL2	ACD, PCD	Symptomatic *Pf* d (19)
	Polley, 2003 [32] a	Upper River	5	334	3–7	MSP-1-BL2	ACD, PCD	Symptomatic *Pf* d (19)
	Metzger, 2003 [33] a	Upper River	5	329	3–7	MSP-2	ACD, PCD	Symptomatic *Pf* d (19)
	Polley, 2007 [34] a	Upper River	5	319	3–7	MSP-3	ACD, PCD	Symptomatic *Pf* d (19)
	Dziegiel, 1993 [35] a	North Bank	6	385	3–8	GLURP	ACD	Symptomatic *Pf* d (35)
	Egan, 1996 [36] a	North Bank	6	327	3–8	MSP-1_19_, MSP-1-EGF	ACD	Symptomatic *Pf* d (35)
	Taylor, 1998 [37] a	North Bank	6	355	3–8	MSP-2	ACD	Symptomatic *Pf* d (35)
	Okenu, 2000 [38] a	North Bank	6	284	3–8	EBA-175	ACD	Symptomatic *Pf* d (35)
	Okech, 2004 [39] a	North Bank	6	260	3–8	MSP-1_19_ b	ACD	Symptomatic *Pf* d (35)
	Gray, 2007 [40] a	North Bank	6	189	3–8	AMA-1, MSP-1_19_,^b^ MSP-1-BL2, MSP-2,^b^ MSP-3	ACD	Symptomatic *Pf* d (35)
**Ghana**	Dodoo, 1999 [41] a	Greater Accra	18	266	3–15	MSP-1_19_,^b^ MSP-1-EGF	ACD, PCD	Symptomatic *Pf* e (41)
	Dodoo, 2000 [42] a	Greater Accra	18	115	3–15	GLURP	ACD, PCD	Symptomatic *Pf* e (41)
	Cavanagh, 2004 [43] a	Greater Accra	18	280	3–15	MSP-1_19_, MSP-1-BL1, MSP-1-BL2	ACD, PCD	Symptomatic *Pf* e (41)
	Dodoo, 2008 [44]	Greater Accra	9	352	3–10	AMA-1, GLURP, MSP-1_19_, MSP-3	ACD, PCD	Symptomatic *Pf* e (19)
**Kenya**	Polley, 2004 [45] a	Coast	6	1,071	0.1–85	AMA-1	ACD, PCD	Symptomatic *Pf* f (15, 26)
	Polley, 2006 [46] a	Coast	6	1,068	0.1–85	MSP-2	ACD, PCD	Symptomatic *Pf* f (15, 26)
	Osier, 2007 [47] a	Coast	6	536	0.1–85	MSP-3	ACD, PCD	Symptomatic *Pf* f (15)
	Osier, 2008 [59] a	Coast	6	280	0.1–85	EBA-175, MSP-1_19_, MSP-1-BL2	ACD, PCD	Symptomatic *Pf* f (24)
**Papua New Guinea**	Al-Yaman, 1995 [48] a	East Sepik	12	230	0.5–15	MSP-2	ACD, PCD	Symptomatic *Pf* e (DNS)
	Al-Yaman, 1996 [49] a	East Sepik	12	230	0.5–15	MSP-1_42_	ACD, PCD	Symptomatic *Pf* e (DNS)
**Senegal**	Perraut, 2005 [50]	Fatick	5	205	3–75	MSP-1_19_	ACD, PCD	Symptomatic *Pf* g (60)
	Sarr, 2006 [60]	Fatick	6	169	2–10	MSP-2	ACD	Symptomatic *Pf* h (53)
**Sierra-Leone**	Egan, 1996 [36]	Southern	12	645	0–8	MSP-1_19_, MSP-1-EGF	ACD	Symptomatic *Pf* d (42)
**Tanzania**	Lusingu, 2005 [51]	Tanga	6	171	0–19	GLURP	ACD, PCD	Symptomatic *Pf* e (32)

Sample size refers to number of participants whose serology was determined. IgG responses measured by ELISA with the exception of Gray et al. [40] who used microarray immunoassays. Manuscripts by Egan et al. [36] and Okech et al. [39] report studies performed in two countries and feature twice in Table 1 and once in Tables 1 and 2, respectively. Studies by Polley et al. [45],[46] in the Kenyan coast were done at two study sites.

a Indicates that the different antibody association studies were performed in the same cohort for the specified country and province. In The Gambia, the “Upper River” and “North Bank” studies were separate cohorts.

b Antigen was not included in meta-analysis (as per protocol).

Malaria definitions:

c History of fever plus *P. falciparum* >300/µl.

d Fever plus *P. falciparum* ≥5,000/µl or fever plus *P. falciparum* >5,000/µl.

e Fever or history of fever (within the past 72 h) plus *P. falciparum* ≥5,000/µl.

f Fever plus an age-dependent threshold of *P. falciparum*.

g Fever plus >30 *P. falciparum* trophozoites/100 leukocytes.

h Fever plus *P. falciparum* >2,500/µl.

*Pf*, *P. falciparum*.

**Table 2:** Characteristics of prospective treatment-to-reinfection studies included in the systematic review by country.

Country	Study: Author, Year [Reference]	Province	Antimalarial	Follow-up (mo)	Population		Merozoite IgG Response	*P. falciparum* Outcome	
					Sample Size	Age (y)		Source	Incidence Outcome (Cumulative Incidence %)
**Kenya**	John, 2004 [52] a	Rift Valley	SP	3	84	1–80	MSP-1_19_	ACD, PCD	Reinfection (45)
	John, 2005 [53] a	Rift Valley	SP	3	84	1–80	AMA-1, EBA-175, *MSP-1_19_* b	ACD, PCD	Reinfection (45)
**Mali**	Tolle, 1993 [54]	Bamako	CQ	7	191	1 to >15	MSP-1-BL2	ACD	High *Pf* density [c] (DNS)
**Papua New Guinea**	Stanisic, 2009 [55]	Madang	A	6	206	5–14	AMA-1, MSP-1_19_, MSP-2	ACD, PCD	Reinfection (95), High *Pf* density [c] (52), Symptomatic *Pf *[d] (49)
**Senegal**	Perraut, 2003 [56]	Fatick	Q	5	110	2–73	MSP-1_19_	ACD	Reinfection (93), Symptomatic *Pf*[e] (66)
**Uganda**	Okech, 2004 [39]	Northern Region	SP	5	156	7–16	MSP-1_19_	ACD	High *Pf* density [c] (18)
**Vietnam**	Wang, 2001 [57]	Khanh-Hoa	Q+D+*P*	6	112	9–55	MSP-1_19_, MSP-4	ACD	Reinfection (42)

Sample size refers to number of participants whose serology was determined. IgG responses measured by ELISA. Okech (2004) [39] performed studies in two countries and also features in Table 1.

a Indicates that the different antibody association studies were performed in the same cohort for the specified country and province.

b Antigen was not included in meta-analysis (as per protocol).

Malaria definitions:

c *P. falciparum* >5,000/µl.

d Fever plus *P. falciparum* ≥5,000/µl or fever plus *P. falciparum* >5,000/µl.

e Fever plus >30 *P. falciparum* trophozoites/100 leukocytes.

A, artesunate; CQ, chloroquine; D, Doxycyline; MSP-1-BL2r, Block 2 repeats; *P*, Primaquine; *Pf*, *P. falciparum*; Q, quinine; SP, sulfadoxine-pyrimethamine.

### Association between Anti-MSP-1 Responses and Incidence of *P. falciparum* Malaria

#### MSP-1 C-terminal (Ct)–processing fragments

**Figure 2:**
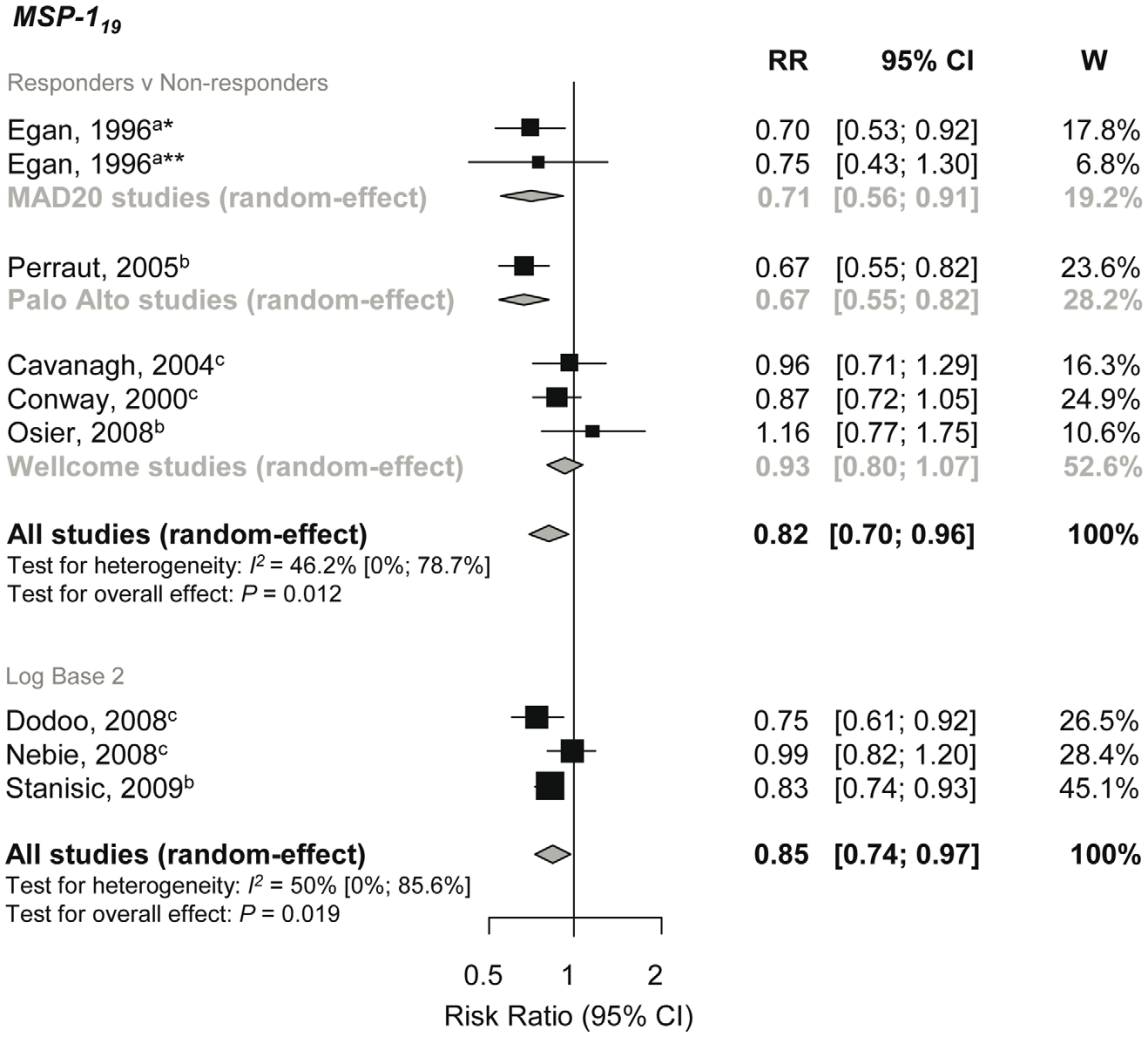
Forest plot of the association of MSP-1_19_ responses with incidence of symptomatic *P. falciparum* malaria. RRs correspond to risk of symptomatic *P. falciparum* malaria for MSP-1_19_ responders versus nonresponders and per doubling of antibody responses (log base 2). RR<1 indicate that antibody responses are protective against symptomatic *P. falciparum* whereas RR>1 indicate susceptibility. ^a^Estimates are calculated by authors from data in the paper; ^b^data supplied by original authors and calculated by current authors; ^c^estimates are published estimates. All estimates are unadjusted with the exception of estimates from Nebie et al. (2008) and Dodoo et al. (2008), which are adjusted for age, and estimates from Stanisic (2009) are adjusted for age and spatial confounders [29],[44],[55]. W, weight. Note: Egan, 1996 had two study sites *Sierra-Leone and **The Gambia, and their analysis only included those with clinical disease versus asymptomatics, i.e., excluded those uninfected as they were assumed to be unexposed [36].

MSP-1 is a high molecular mass protein (Mr≈180 kDa) that is proteolytically processed into 83 kDa, 30 kDa, 38 kDa, and C-terminal 42 kDa (MSP-1_42_) fragments [61]. During invasion, MSP-1_42_ is further processed into MSP-1_19_ and MSP-1_33_ fragments. Both MSP-1_19_ and MSP-1_42_ are regarded as potential vaccine candidates and have been shown to be protective in animal models [17]. Meta-analysis of five studies showed that MSP-1_19_ IgG responders had an 18% reduction in the risk of symptomatic *P. falciparum* malaria compared to nonresponders (pooled RR using random-effects [reRR] 0.82, 95% CI 0.7–0.96, *p* = 0.012; Figure 2) [31],[36] [43] [50] [59]. Meta-regression analysis revealed heterogeneity between allelic groups (*p* = 0.0223) with the greatest magnitude of effect seen with MAD20 and Palo Alto alleles (29% and 33% relative reduction in symptomatic disease, respectively; Figure 2). Because the methods for the preparation of each antigen was the same for each allelic variant (Text S2, Table A) similar results were obtained when grouping according to expression system and tag used to make the recombinant antigen. Other methodological and clinical characteristics of the studies did not influence estimates and there was no evidence of publication bias.

Data were obtained from a further three studies and pooled to examine the dose-response association of MSP-1_19_ levels (log base 2) and the risk of malaria [29],[44],[55]. A 15% reduction in symptomatic *P. falciparum* per doubling of antibody levels was observed (reRR 0.85, 95% CI 0.74–0.97, *p* = 0.019; Figure 2). With only three studies in the meta-analysis, further subgroup analysis was not feasible. One additional study examined the association of antibody levels (excluded from meta-analysis because transformation, if any, was not stated in the original manuscript) with symptomatic *P. falciparum* and found weak evidence of a protective effect (RR 0.97, 95% CI 0.94–1.00, *p* = 0.0713) [56]. There was no conclusive evidence to support an association between anti-MSP-1_19_ responses with *P. falciparum* high density infection or reinfection (see Text S2).

MSP-1_19_ is made up of two epidermal growth factor-like modules (EGF-1 and EGF-2). Meta-analyses showed no association between the presence of responses to either MSP-1-EGF1 or MSP-1-EGF2 with protection against symptomatic *P. falciparum* (RR 1.06, 95% CI 0.88–1.26, *p* = 0.56 and reRR 0.59, 95% CI 0.19–1.84, *p* = 0.37; *I* ^2^ = 71.4%, 95% CI 2.8–91.6%, respectively) [36],[41]. For individual study estimates see Text S2.

Only one study examined the association of MSP-1_42_ levels (log base 2) with incidence risk of symptomatic *P. falciparum* and found a reduced risk (RR 0.76, 95% CI data not shown in original manuscript [DNS], *p*≤0.001) [49].

#### MSP-1 polymorphic N-terminal regions

**Figure 3:**
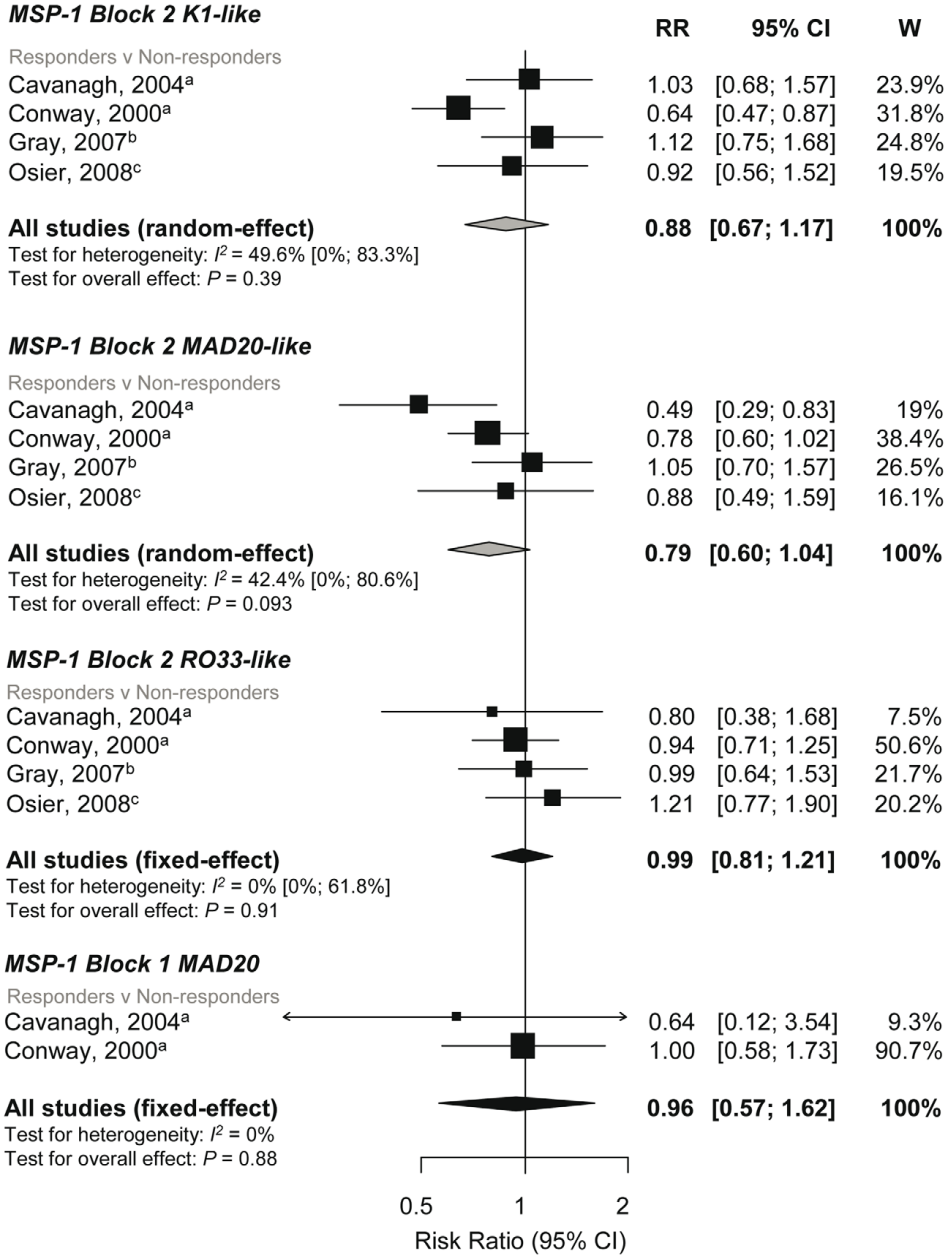
Forest plot of the association of MSP-1 block 2 and block 1 responses with incidence of symptomatic *P. falciparum* malaria. RRs represent the risk of symptomatic *P. falciparum* malaria in IgG responders relative to nonresponders. RR<1 indicate that responders are protected from symptomatic *P. falciparum* whereas RR>1 indicate susceptibility. ^a^Estimates are published estimates; ^b^estimates are calculated by authors from data in the paper; ^c^data supplied by original authors and calculated by current authors. All reported estimates are unadjusted. W, weight.

MSP-1 block 2 can be grouped into three allelic types, K1-like, RO33-like, and MAD20 like. The association of incidence risk of symptomatic *P. falciparum* with allelic specific MSP-1 block 2 responders compared to nonresponders was examined in four studies [31],[40],[43],[59]. Pooled results were done separately for each allelic type. Meta-analysis revealed no evidence of an association with the K1-like (reRR 0.88, 95% CI 0.67, 1.17, *p* = 0.39) or RO33-like allele (RR 0.99, 95% CI 0.81, 1.21, *p* = 0.91) (Figure 3). There was weak evidence of a protective effect of MAD20-like responses with incidence risk of symptomatic *P. falciparum* (reRR 0.79, 95% CI 0.6, 1.04, *p* = 0.093; Figure 3).

**Figure 4:**
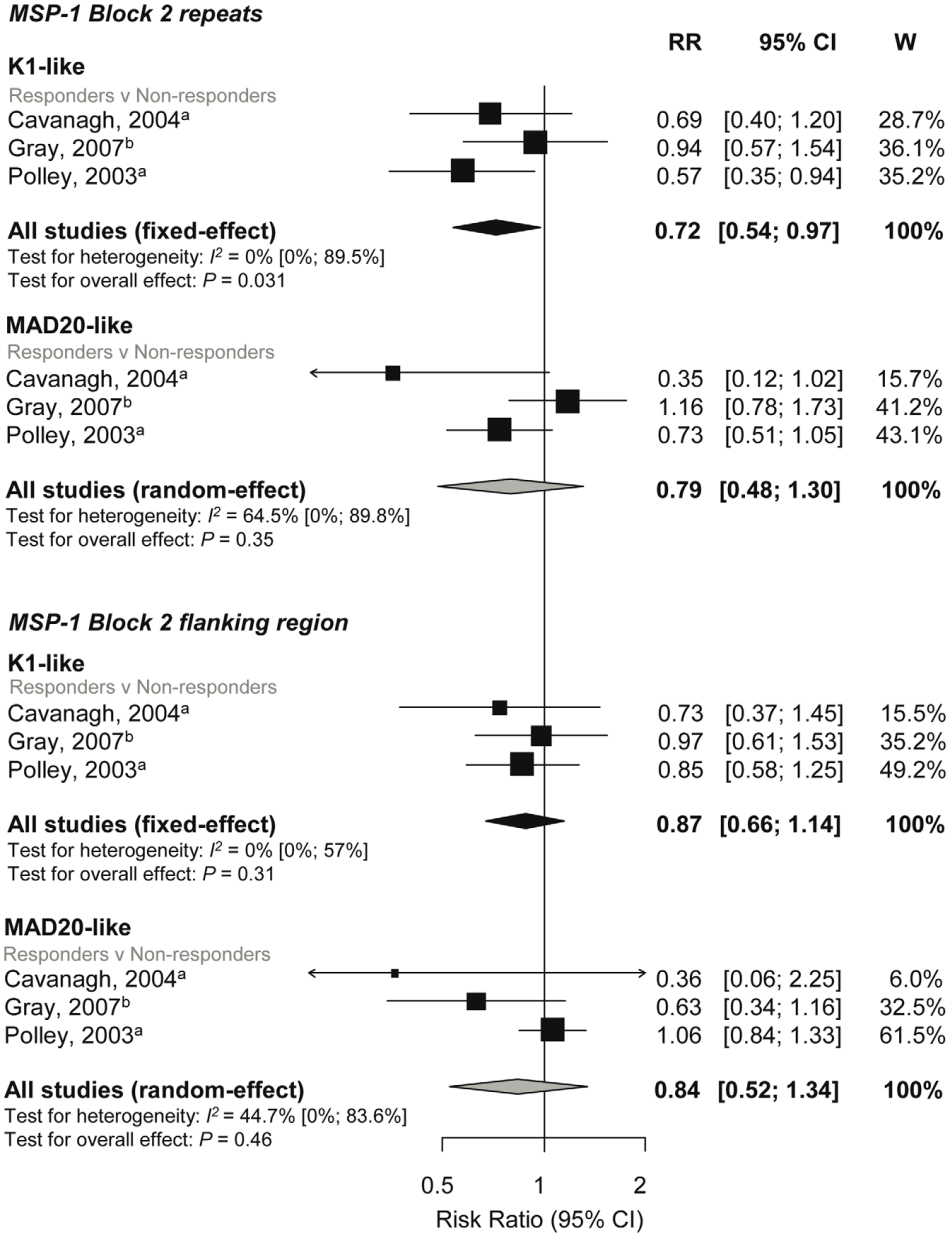
Forest plot of the association of MSP-1-block 2 repeats and flanking region responses with incidence of symptomatic *P. falciparum* malaria. RRs represent the risk of symptomatic *P. falciparum* malaria in IgG responders relative to nonresponders. RR<1 indicate that responders are protected from symptomatic *P. falciparum* whereas RR>1 indicate susceptibility. ^a^Estimates are published estimates; ^b^estimates are calculated by authors from data in the paper. All reported estimates are unadjusted. W, weight.

The K1-like and MAD20-like types of MSP-1 block 2 differ in the length of tri-peptide repeats in the middle of the block as well as the flanking nonrepetitive sequences. Meta-analysis was performed on three studies investigating the association between responses to MSP-1 block 2 repeats and flanking regions (responders versus nonresponders) and incidence risk of symptomatic *P. falciparum* [32],[40],[43]. There was some evidence of an association for MSP-1 block 2 K1-like repeats (RR 0.72, 95% CI 0.54–0.97, *p* = 0.031) but not MSP-1 block 2 MAD20-like repeats (reRR 0.79, 95% CI 0.48–1.3, *p* = 0.35) (Figure 4). There was also no evidence of an association between MSP-1 block 2 flanking regions with risk of symptomatic *P. falciparum* (K1-like RR 0.87, 95% CI 0.66–1.14, *p* = 0.31 and MAD20-like reRR 0.84, 95% CI 0.52–1.34, *p* = 0.46; Figure 4).

Combined results from two studies showed no evidence of an association of MSP-1 block 1 responses with risk of symptomatic falciparum malaria (responders versus nonresponders RR 0.96, 95% CI 0.57–1.62, *p* = 0.88; Figure 4) [31],[43].

### Association between Anti-MSP-2 Responses and Incidence of *P. falciparum* Malaria

**Figure 5:**
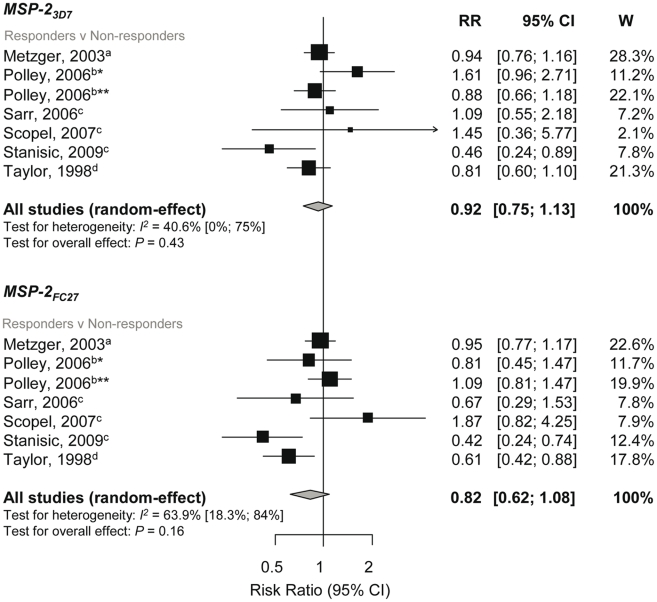
Forest plot of the association of MSP-2 responses with incidence of symptomatic *P. falciparum* malaria. RR<1 indicate that responders are protected from symptomatic *P. falciparum* compared to nonresponders whereas RR>1 indicate susceptibility. ^a^Estimates are published estimates; ^b^converted published estimate; ^c^estimates are calculated by authors from data supplied by original author; ^d^estimates are calculated by authors from data in the paper. W, weight. Estimates reported are unadjusted with the exception of Stanisic (2009) (adjusted for spatial confounders and age) and Metzger (2003) (adjusted for age and preseason parasitaemia) [33],[55]. Note that estimates for Taylor (1998) are based on clinical and asymptomatic cases only (i.e., those uninfected were excluded on the basis they were unexposed) [37]. Polley (2006) stratified for two study sites in Coastal Kenya, *Chonyi and **Ngerenya [46].

The single *msp2* locus of *P. falciparum* is highly polymorphic but can be grouped into two major allelic types, 3D7 and FC27. Meta-analysis of six studies investigating MSP-2_3D7_ and MSP-2_FC27_ showed no evidence of a reduced risk of symptomatic *P. falciparum* in those with responses compared to those without responses (MSP-2_3D7_, reRR 0.92, 95% CI 0.75–1.13, *p* = 0.43; MSP-2_FC27_, reRR 0.82, 95% CI 0.62–1.08, *p* = 0.16, Figure 5) [33],[37],[46],[55],[58],[60]. Methodological and clinical characteristics of the studies did not influence estimates and there was no evidence of publication bias. One additional study found a dose-dependent response with MSP-2_3D7_ antibody levels (log base 2) and risk of symptomatic *P. falciparum* (RR 0.81, 95% CI DNS, *p* = 0.003) but not MSP-2_FC27_ (RR 0.99, 95% CI DNS, *p* = 0.86) [48]. Another study examined the effect of MSP-2-Ct (responders versus nonresponders) and found no evidence of an association with symptomatic *P. falciparum* (RR 0.55, 95% CI 0.27, 1.14, *p* = 0.11) [33]. Only one study examined the association of MSP-2 antibodies with reinfection and high density infection and found no association (see Text S2) [55].

### Association between Anti-MSP-3 Responses and Incidence of *P. falciparum* Malaria

**Figure 6:**
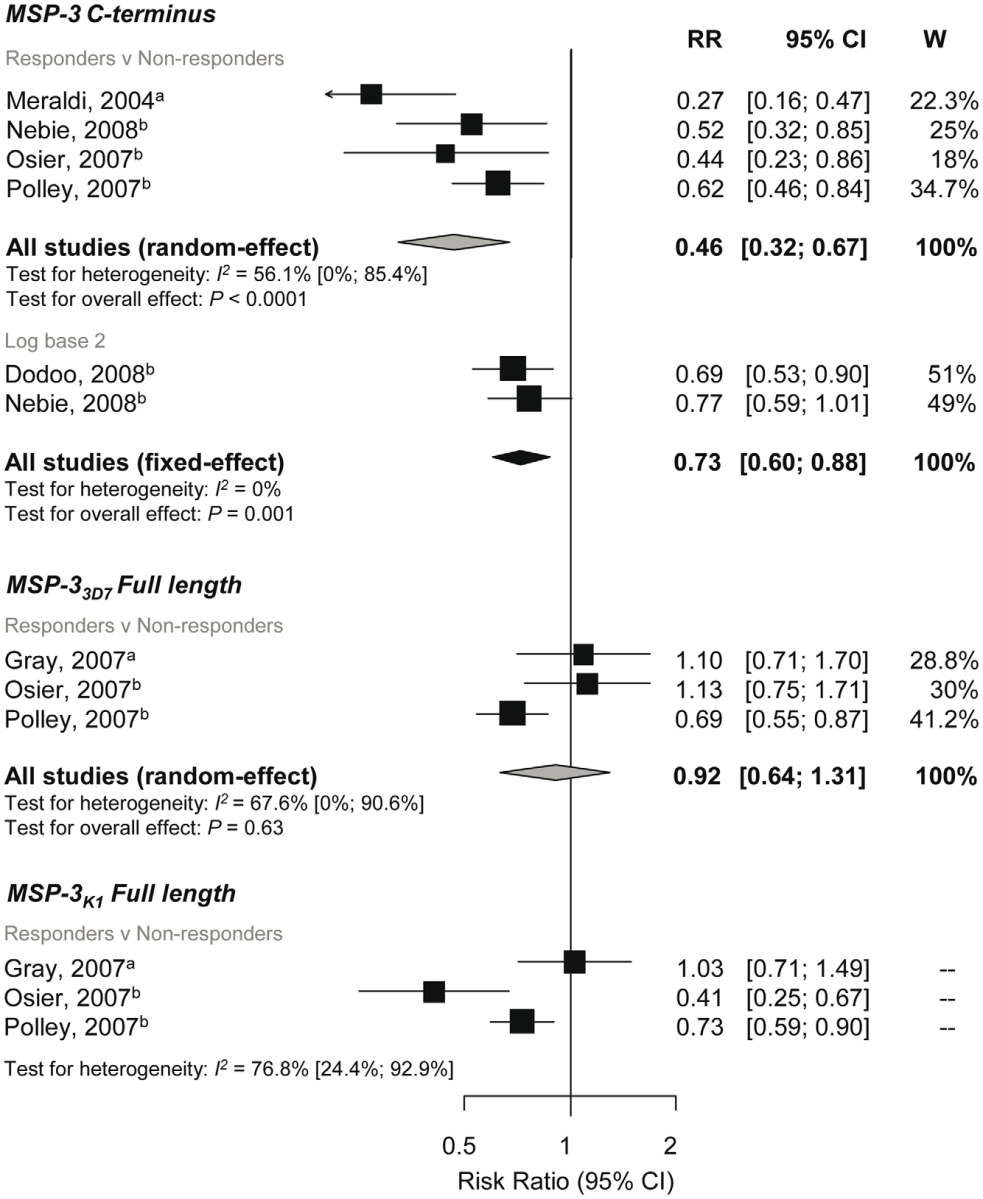
Forest plot of the association of MSP-3 responses with incidence of symptomatic *P. falciparum* malaria. RR<1 indicate protection from symptomatic *P. falciparum* whereas RR>1 indicate susceptibility in responders versus nonresponders or per doubling of antibody responses. Estimates reported are unadjusted with the exception of Nebie (2008) (adjusted for age, sex, and village) [30] and Nebie (2008) and Dodoo (2008) (adjusted for age) [29],[44]. ^a^Estimates are calculated by authors from data in the paper; ^b^estimates are published estimates. All reported estimates are unadjusted. W, weight.

The C-terminal region of MSP-3 (MSP-3-Ct) is highly conserved whereas the remainder of the sequence is defined by two major allelic types, 3D7 and K1 [62]. Meta-analyses of four studies [28],[30],[34],[47] examining antibodies to MSP-3-Ct responses showed a 54% reduction in symptomatic *P. falciparum* in responders versus nonresponders (RR 0.46, 95% CI 0.32–0.67, *p*<0.0001; Figure 6). Meta-analyses of two studies also showed a decreased incidence risk per doubling of MSP-3-Ct antibody levels (RR 0.73, 95% CI 0.6–0.88, *p* = 0.001, Figure 6) [29],[44].

Three studies examined the association of full length MSP-3_3D7_ and MSP-3_K1_ responses (responders versus nonresponders) with risk of symptomatic *P. falciparum* [34],[40],[47]. Meta-analysis showed no evidence of an association with anti-MSP-3_3D7_ responses (reRR 0.92, 95% CI 0.64–1.31, *p* = 0.63; Figure 6), but a large amount of heterogeneity was observed (*I* ^2^ = 67.6%, 95% CI 0–90.6%). A high degree of heterogeneity was also seen for MSP-3_K1_ associations (*I* ^2^ = 76.8%, 95% CI 24.4–92.9) so results were not combined (Figure 6). Due to the small number of studies in these meta-analyses, exploration of heterogeneity by subgroup analysis was not feasible.

### Association between anti-AMA-1 Responses and Incidence of *P. falciparum* Malaria

**Figure 7:**
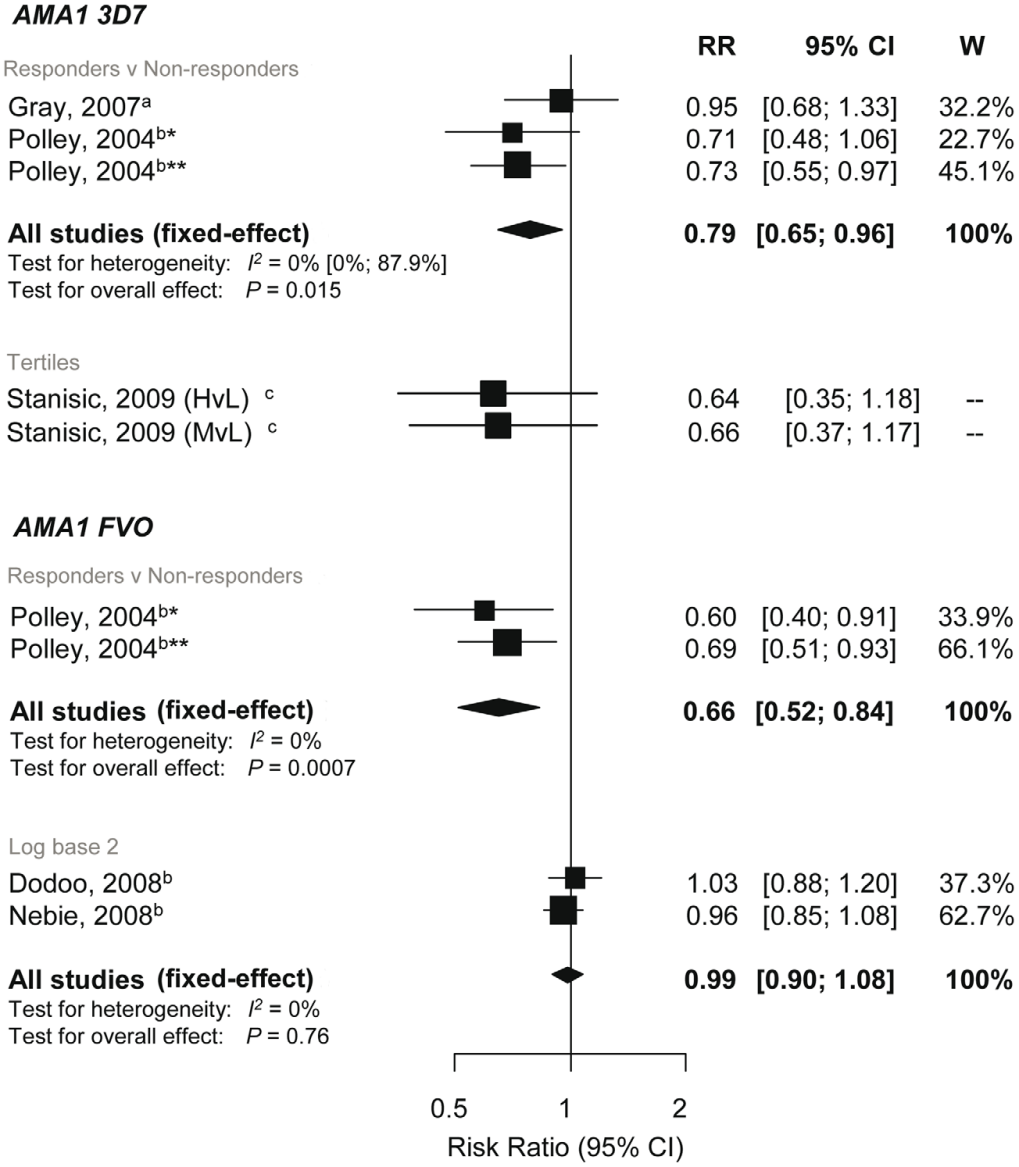
Forest plot of the association of AMA-1 responses with incidence of symptomatic *P. falciparum* malaria. RRs correspond to risk of symptomatic *P. falciparum* malaria for AMA1 responders versus nonresponders, High (H) and medium (M) versus low (L) responders (based on tertiles because sero-prevalence was high) and per doubling of antibody responses (log base 2). RR<1 indicate that antibody responses are protective against symptomatic *P. falciparum* whereas RR>1 indicate susceptibility. ^a^Estimates are calculated by authors from data in the paper; ^b^estimates are published estimates; ^c^estimates supplied by the original authors. All estimates are unadjusted with the exception of Dodoo (2008) and Nebie (2008) with adjustments for age and Stanisic (2009) with adjustments for age and spatial confounders [29],[44],[55]. Polley (2004) stratified for two study sites in Coastal Kenya, *Chonyi and **Ngerenya.

There are currently two different AMA-1 strains of the full-length ectodomain under development as vaccine candidates (FVO and 3D7) [17]. There was evidence of reduced risk of symptomatic *P. falciparum* with AMA-1_3D7_ responders versus nonresponders (RR 0.79, 95% CI 0.65–0.96, *p* = 0.015), and there was also a tendency towards a protective effect in the study that examined tertiles (Figure 7) [40],[45],[55]. For AMA-1_FVO_, one study showed a reduced risk of symptomatic *P. falciparum* in AMA-1_FVO_ responders compared to nonresponders (RR 0.66 95% CI 0.52–0.84, *p* = 0.0007), but combined results of two studies showed no association of anti-AMA-1_FVO_ levels (log base 2) with incidence risk of symptomatic *P. falciparum* (RR 0.99, 95% CI 0.9–1.08, *p* = 0.76; Figure 7) [29],[44],[45]. There was insufficient evidence to show an association between AMA-1 responses with risk of reinfection and high density *P. falciparum* (see Text S2).

### Association between Anti-GLURP Responses and Incidence of *P. falciparum* Malaria

**Figure 8:**
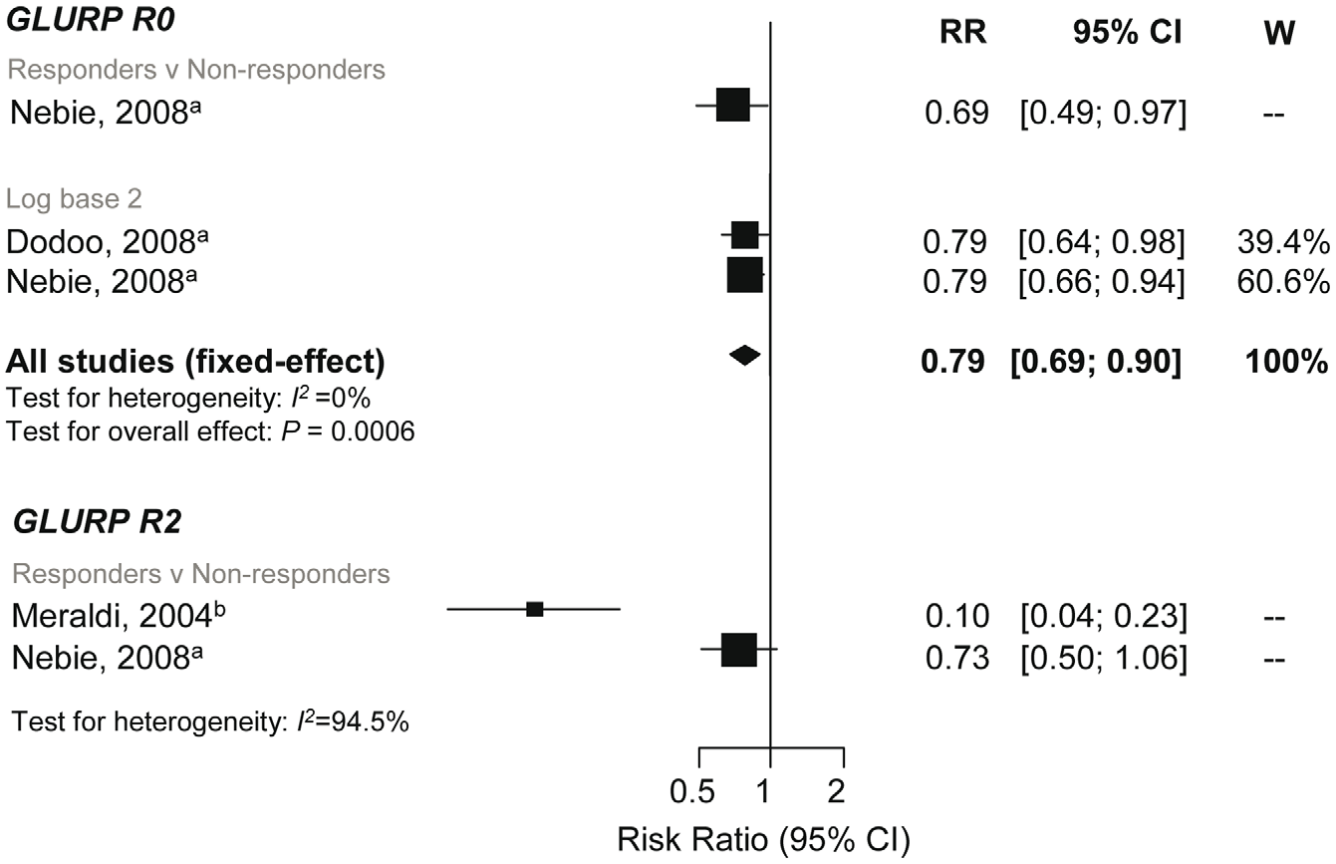
Forest plot of the association of GLURP responses with incidence of symptomatic *P. falciparum* malaria. RRs correspond to risk of symptomatic *P. falciparum* malaria for GLURP responders versus nonresponders and per doubling of antibody responses (log base 2). RR<1 indicate that antibody responses are protective against symptomatic *P. falciparum* whereas RR>1 indicate susceptibility. ^a^Estimates are published estimates with adjustments for age, Nebie (2008) responder versus nonresponder analysis also adjusted for sex and village [30]; ^b^estimates are calculated by authors from data in the paper. GLURP-R2 estimates were not combined because *I* ^2^>75%. W, weight.

GLURP can be divided into an N-terminal nonrepeat region (R0), a central repeat region (R1), and a C-terminal repeat region (R2). A reduced risk of symptomatic *P. falciparum* was shown in GLURP-R0 responders compared to nonresponders (RR 0.69, 95% CI 0.48–0.97, *p* = 0.032) and per doubling of antibody levels (RR 0.79, 95% CI 0.69–0.91, *p* = 0.0006; Figure 8) [29],[30],[44]. Dodoo et al. (2000) also reported that anti-GLURP-R0 levels were associated with protection (*p*<0.005), but no estimates or 95% CI were given [42]. Conversely, Lusingu et al. (2005) reported no association with anti-GLURP-R0 responders with odds (RR were incalculable) of symptomatic episode (OR 1.13, 95% CI 0.5–2.53, *p* = 0.77) [51].

GLURP-R2 was associated with protection to varying degrees. Meraldi et al. and Nebie et al. showed a 90% (RR 0.1, 95% CI 0.05–0.23, *p*<0.001) and 27% (RR 0.73, 95% CI 0.5–1.06, *p* = 0.1; Figure 8) reduction in symptomatic malaria in responders versus nonresponders [28],[30]. Estimates from these two studies were not combined (*I* ^2^ = 94.5%). Another study found no evidence of an association between anti-GLURP-R2 (*p* = 0.2) or GLURP-R1 (*p* = 0.3) levels (estimates and 95% CI, DNS) [42]. One study examined the association of GLURP-R1-R2 with malaria, which showed a reduced risk of symptomatic *P. falciparum* (RR 0.73, 95% CI 0.55–0.97, *p* = 0.03) [35].

### Association between Other Responses and Incidence of *P. falciparum* Malaria

Only three studies meeting our inclusion and quality criteria measured anti-EBA-175 responses [38],[53],[59]. Osier et al. (2008) (used recombinant F2 domain) and Okenu et al. (2000) (used recombinant region II) showed no association of anti-EBA-175 antibodies with risk of symptomatic *P. falciparum* (responders versus nonresponders, RR 1.36, 95% CI 0.81–2.3, *p* = 0.246 for Osier et al.; RR 0.96, 95% CI 0.71–1.29, p = 0.77 for Okenu et al.). John et al. (2005) showed no association between antibodies (to region II) and risk of reinfection (>75 percentile versus <75% percentile 1.25, 95% CI 0.66–2.36, *p* = 0.49). One study investigated the relationship between MSP-4 and MSP-4-EGF1 with risk of reinfection and showed no association (RR 0.9, 95% CI 0.58–1.39, *p* = 0.64 and RR 0.8, 95% CI 0.56–1.15, *p* = 0.22, respectively) [57].

## Discussion

This systematic review strongly supports the protective effect of total IgG responses to particular merozoite surface antigens against symptomatic *P. falciparum* malaria in humans. Meta-analyses showed that individuals who have IgG to MSP-3-Ct and MSP-1_19_ have a risk of symptomatic *P. falciparum* that is 54% and 18%, respectively, less than those without detectable IgG. Moreover, there was evidence of a dose-response relationship such that the magnitude of association with these antigens increased per doubling of antibody levels. A tendency towards protective RR was also observed when individual estimates for AMA-1_3D7_ and GLURP-R0 were examined, but pooled estimates of more than two studies could not be determined owing to heterogeneity among studies. Pooled estimates showed limited evidence of a protective effect of IgG responses towards MSP-2, MSP-1 N-terminal region, or MSP-1-EGF subregion with symptomatic *P. falciparum* malaria. Importantly, this systematic review revealed a paucity of studies examining the association of IgG responses towards the vaccine candidates MSP-1_42_ and EBA-175 with incidence of *P. falciparum* malaria.

Heterogeneity, in terms of both clinical and methodological diversity between studies, was an important issue in the meta-analyses. Clinical heterogeneity was noted in MSP-1_19_ meta-analyses whereby the magnitude of effect varied with allelic group. However, given that MSP-1_19_ is relatively conserved and that the different alleles are based on four amino acid changes, the biological relevance of this observation is unknown. Methodological heterogeneity was most evident across studies investigating AMA-1 and GLURP responses. Antibody variables were defined differently across studies and estimates with errors and/or raw data were not presented. Subsequently the standardization of antibody variables and pooling of results was problematic. Statistical heterogeneity (*I* ^2^ value) was greatest for GLURP R2 and the full length MSP-3 antigen meta-analyses.

There are many factors influencing the selection of antigens for vaccine development and testing in clinical trials, and evidence from observational studies can provide valuable knowledge to inform this process. MSP-1_19_ was the most featured merozoite surface antigen and meta-analyses showed that antibody responses to MSP-1_19_ were indicative of protection. It is thus surprising that MSP-1_19_ has only featured in one vaccine in humans, in which it was used in combination with AMA-1 (PfCP2.9/ISA720) in phase I trials [63],[64]. Conversely, only one study has demonstrated evidence of protection for antibodies to MSP-1_42_, but this antigen has been tested in a phase II vaccine trial where it was not protective [65]. The reasons for the failure of this vaccine remain unclear, but may relate to antigen polymorphism or the nature of the vaccine-induced response, or instead may indicate that MSP-1_42_ antibodies are not protective. Further studies of this antigen are clearly needed. Other merozoite surface antigens currently undergoing phase II trials in malaria endemic countries include AMA-1 (AMA-C1, which includes 3D7 and FVO strains) and MSP-3 (as a long synthetic peptide and a MSP-3/GLURP chimera), which were shown to be protective against symptomatic malaria in this review [17]. There are currently no vaccines with MSP-1-block 1 and 2 proteins, and data from this systematic review does not support the development of these antigens as vaccine targets.

The aim of this systematic review was to be as comprehensive and inclusive as possible and fulfil guidelines for meta-analyses [19]. We performed an extensive search of six different databases and did not limit our searches by language to remove the potential for bias due to exclusion of non-English studies [66]. Furthermore, we identified and contacted the investigators for the studies that did not meet our initial inclusion and quality criteria but contained potential data. In addition, instead of excluding studies that did not provide estimates, we contacted authors and asked them to provide estimates or data. We did not limit our review to IgG subclasses as it would substantially decrease the number of studies included. Examining subclass-specific responses to merozoite antigens has provided further insights into protective targets and mechanisms of acquired immunity [55],[67]. However, differences in the specificity and sensitivity of subclass-typing reagents between studies makes comparisons between studies difficult. We also assessed publication bias where possible, although in some cases where only a few studies were combined this assessment was difficult. The extent to which the selective publication of studies based on the direction and magnitude of findings within malaria epidemiological research is unknown. The publication of all studies regardless of findings should be encouraged.

Determining a causal relationship between antibodies and protection against *P. falciparum* malaria is one of the main challenges in malaria immuno-epidemiology. Study designs used in the published literature include cross-sectional studies, case-control studies, and cohort studies. To ensure the best inference of causality from the published literature we did two things. Firstly, we only included studies that examined the association of antibodies with prospectively collected *P. falciparum* data to establish a temporal relationship between antibody responses and risk of *P. falciparum* malaria. Secondly, we included a parasitaemia density cut-off in our definition of symptomatic malaria as part of our quality criteria to improve specificity and ensure that *P. falciparum* was the causative agent of the febrile episode. The prevailing view in the field is that a cut-off level of parasitaemia is needed to improve the specificity of clinical malaria diagnosis in most populations [68]–[70]. The population-specific definitions of a high *P. falciparum* density cut-off in the studies ranged from >300 parasites/µl to >5,000 parasites/µl and the sensitivity of these definitions would vary across populations. In addition, we would expect reduced specificity of the definition for the one study that reanalysed data with a high density cut-off for inclusion in our review [58].

A causal relationship between anti-merozoite antibodies and *P. falciparum* malaria is strengthened by the consistent demonstration of findings under different circumstances. Consistent findings were demonstrated for some antigens despite differences in the preparation of antigens, malaria endemicity, study participants, and study area. Interestingly, we found very few published studies that were performed outside Africa. Of the 32 included studies, only one was performed in Asia (excluding Papua New Guinea) and only one in South America (see Table 1). The generalizability of our findings to populations living in these less-represented regions is unknown. Additionally, we only identified two studies that investigated allele-specific immunity (both studies MSP-2 only), whereby the allele-specific antibody response was related to the strain causing the malaria episode [55],[58]. If protection is purely allele-specific then the true causal protective effect will be underestimated in studies that do not use allele-specific *P. falciparum* outcomes.

Another important limitation in published literature is that data generated by ELISA does not produce a common metric measurement thereby restricting the standardization of exposure variables. In meta-analyses we were able to pool RR for responders versus nonresponders and RR derived from log base 2 antibody levels, which represent the change in risk per doubling of antibody levels. However, antibody concentrations vary across populations according to the level of exposure to malaria. Therefore the magnitude of effect according to quantified responses may vary significantly across studies. This was evident by the dose-dependent relationships between some antibody responses and level of protection and would suggest that antibody responses need to be quantified. Furthermore, knowledge on how long specific merozoite antibody responses last, how they are boosted, and the duration of any protection from responses is presently limited. The duration of the follow up in observational studies may therefore have an impact on the strength and direction of an association, an effect we explored in meta-regression. Further studies that measure responses at multiple time points are needed to better understand these issues.

The definition of “protected” individuals (i.e., those who did not have symptomatic malaria) varied across studies. For most studies this definition included all participants who had no recorded episodes of symptomatic *P. falciparum* malaria. Three studies excluded individuals who did not have any detected parasitaemia during follow-up from the “protected” group, on the basis that these individuals were unexposed [35]–[37]. Only the six treatment-to-reinfection studies had regular blood collection for detection of parasitaemia; all other included studies only collected blood slides during follow-up when an individual was febrile, so accurately determining true “unexposed” individuals in areas where asymptomatic parasitaemia is prevalent will be problematic. Recent analyses by Bejon et al. (2009) of anti-VSA antibodies in individuals living in Kilifi, Kenya, showed that by removing unexposed children from conventional analyses, the magnitude of effect was greater between those with high and low responses [71]. This is consistent with other studies in Kilifi that showed that associations between specific merozoite antibody responses and protection were stronger in children who were asymptomatic at baseline [45],[46],[59]. A further consideration is that studies in malaria-endemic areas typically compare individuals with different levels of immunity, not individuals with complete immunity versus individuals with no immunity. Therefore, the reported effect size may not accurately reflect the true magnitude of the response in the study population.

### Conclusion and Guidelines for Future Research

IgG responses to some, but not all, merozoite surface antigens were associated with protection against symptomatic *P. falciparum* in malaria endemic areas. We identified very few antigens that had been well studied and a deficiency of studies done outside Africa. More studies in different populations, examining multiple antigens at multiple time-points, are needed to better determine the role of anti-merozoite antibodies in protection against malaria, with prospective cohort studies as the preferred study design to establish temporal causality. In the future, there should be as much uniformity between studies as possible to ensure maximum comparability. This could be improved by the quantification and standardization of IgG responses, which could be achieved by establishing a reference reagent for determining antibody concentrations. Furthermore, the protective effects of anti-merozoite responses observed epidemiologically must also be supported by evidence of the function of the antibodies. Development and application of functional assays rather than standard immunoassays would also be highly valuable. Presently, data on the function of antibodies against merozoite antigens is very limited [8],[12],[15],[72]. Lastly, there is a need to incorporate strain-specific responses and endpoints to address whether protective responses against particular antigens are strain-transcending or strain-specific.

A challenging aspect of this systematic review was the standardization of exposure and outcome measurements as there is no consistent approach to reporting of data. To facilitate the standardization of results in future studies, we propose guidelines for the reporting of malaria immuno-epidemiology studies adapted from the Strengthening the Reporting of Observational Studies in Epidemiology (STROBE) statement (Table 3) [73],[74]. Standardizing studies, and removing as much methodological heterogeneity as possible, will help obtain more comparable results in the future. By doing so, we will then be in a more favourable position to assess the relative contribution of responses to certain antigens, thereby informing vaccine candidate choices.

**Table 3:** Proposed guidelines of the reporting of Malaria Immuno-epidemiology Observational Studies (MIOS guidelines).

Report Section	Topics	Recommended Inclusions
**Title and abstract**	**—**	Indicate the study design and the study population
	**—**	Provide in the abstract an informative and balanced summary of what was done and the main findings. Indicate immune response measured, antigens used, and all *Plasmodium* and clinical end-points examined. Present key estimates of associations with measures of variability.
**Introduction**	**—**	Explain the scientific background and rationale for the antigens and *Plasmodium* end-points chosen.
	**—**	State objectives, including any prespecified hypotheses (i.e., protection, no effect).
	**—**	State how the current study will add to the malaria immuno-epidemiology literature and briefly state how it compares to previous studies.
**Methods**	**Epidemiological study**	A description of the setting, including location, *Plasmodium spp.* found in the area, rate of malaria transmission, dates of transmission. Mention any recent changes in endemicity.
	**—**	Study design, describe exactly how and when immune response, *Plasmodium* and clinical data collection took place. For longitudinal studies discriminate between serial cross-sectional studies and longitudinal cohort studies.
	**—**	Relevant dates such as participant recruitment, measurement of immune responses, follow-up, and *Plasmodium* and clinical data collection.
	**—**	Eligibility criteria and sources and methods of selection of participants. Justification of criteria.
		Methods of follow-up and data collection. Indicate intervals for ACD and the appropriateness of the use of PCD in the setting. Indicate how presumptive malaria diagnosis was dealt with in data collection.
	**—**	A description of any efforts to address potential sources of bias.
	**—**	Sample size calculations. Include the level of precision and power, the expected size of differences to be measured (e.g., in antibody levels, risk/odds of malaria), and the minimum difference you wish to detect.
	**Variables**	Definitions of all *Plasmodium* outcomes (i.e., parasitaemia, symptomatic malaria), detail parasitological cut-offs and fever definitions. State whether *Plasmodium* speciation was done and how this was incorporated into definitions. Mention the sensitivity and specificity of malaria definitions in the population. Indicate how “unexposed” individuals were defined, if relevant.
	**—**	Definitions of all immunological variables. Explain how responders and nonresponders were defined. Explain how continuous variables were handled in the analyses such as the use of transformations and groupings. Describe which groupings were chosen and why, and state the cut-offs used for each group and the category mean or median values. For each antigen indicate the allele, amino acid position, expression system, and tag. Provide gene accession numbers.
	**—**	A list of all potential confounders and effect modifiers that were considered with justification. These should at least include age, *Plasmodium* status at baseline, and variables that represent level of transmission/exposure (e.g., spatial confounders).
	**Statistical analysis**	Rationale for statistical approach considering study design and distribution of immunological and *Plasmodium* data. Make particular note of any collinearity issues with immunological data.
	**—**	Description of all statistical methods, including those used to control for confounding, examine subgroups and interactions (particularly with age) and any sensitivity analyses. Explain how missing data were addressed if relevant.
	**—**	Details and justification of all data transformations explored during analysis. State any assumptions of linearity in immunological data. State whether categories generated from continuous antibody variables were used as a nominal or ordinal variable (i.e., classified into unordered or ordered qualitative categories).
**Results**	**Study participants**	The numbers of individuals at each stage of the study and any groups excluded from analysis.
	**—**	The demographic and clinical characteristics of the participants and information on exposures and potential confounders. Indicate the number of participants with missing data for each variable of interest. Summarize follow-up times if applicable and mention changes in incidence of *Plasmodium* over follow-up. Consider presenting clinical and immunology data according to age group to give the reader a sense of the acquisition of immunity in the study population or by immunological response categories so they can be related to confounders.
	**Immunological responses and malaria measures**	Mean (standard deviation) or median (percentiles/range) of values to describe measures of central tendency and the spread of data measured in the study. Do not use inferential measures such as standard errors or confidence intervals.
	**—**	Details of any quantification of antibody or other concentrations (i.e., titres in µg/ml).
	**—**	Counts of cases, controls, person-time at risk, risk etc. for each immune response category in addition to effect-measure estimates and results of model fitting.
	**Risk estimates**	Unadjusted and adjusted estimates of risk and their precision, e.g., 95% CIs. This will allow the reader to judge by how much, and in what direction, they changed. Make clear which confounders were adjusted for and why they were included. Provide risk estimates for all immunology variables investigated (i.e., responders versus nonresponders and any dose-dependent variables).
	**—**	Separate estimates for each immune response. Also assess joint effects and interactions between immune responses. Consider both additive and multiplicative scales (i.e., does the combined effect of response *A* and *B* add (*a*+*b*)% or (*a*×*b*)% to risk?). This will help assess the relative contribution of each immune response to protection.
	**—**	Separate estimates for different lengths of follow-up. E.g., 1, 3, 6, 9, 12 mo.
	**—**	Report all other analyses done such as subgroups, interactions, and sensitivity analysis.
**Discussion**	**—**	Summarise key results in relation to study objectives
	**—**	Provide limitations of your study.
	**—**	Give a balanced interpretation of the results considering limitations. Discuss both direction and magnitude of effects and pay particular attention to evidence of no effect versus no evidence of an effect. Outline possible methodological reasons for why the current results may differ from other studies.
	**—**	Discuss the generalisability of results to other malaria endemic areas.

Items should be addressed in the main body of the manuscript and/or supplementary material. This table has been adapted from the Strengthening the Reporting of Observational Studies in Epidemiology (STROBE) statement, which contains a checklist of items that should be addressed in reports of observational studies [74]. The STROBE statement and explanation [73],[74] should also be consulted.

## Supporting Information

Text S1 PRISMA checklist.
(0.07 MB DOC)

Text S2 Excluded studies, supplementary tables, and analyses.
(0.54 MB DOC)

## Abbreviations

ACD: active case detection
AMA-1: apical membrane antigen-1
BL: block
CI: confidence interval
Ct: C terminus
DNS: data not shown in original manuscript
EBA: erythrocyte binding antigen
EGF: epidermal growth factor-like module
ELISA: enzyme-linked immunosorbent assay
GLURP: glutamate-rich protein
HR: hazard ratio
Ig: immunoglobulin
IRR: incidence rate ratio
ln: natural logarithm
MSP: merozoite surface protein
OR: odds ratio
PCD: passive case detection
reRR: pooled risk ratio using random-effects
RR: risk ratio
VSA: variant surface antigen

## References

[1] Marsh K, Kinyanjui S (2006). Immune effector mechanisms in malaria.. Parasite Immunol.

[2] Cohen S, Mc GI, Carrington S (1961). Gamma-globulin and acquired immunity to human malaria.. Nature.

[3] Sabchareon A, Burnouf T, Ouattara D, Attanath P, Bouharoun-Tayoun H (1991). Parasitologic and clinical human response to immunoglobulin administration in falciparum malaria.. Am J Trop Med Hyg.

[4] Cohen S, Butcher GA, Crandall RB (1969). Action of malarial antibody in vitro.. Nature.

[5] Brown GV, Anders RF, Mitchell GF, Heywood PF (1982). Target antigens of purified human immunoglobulins which inhibit growth of *Plasmodium falciparum* in vitro.. Nature.

[6] McCallum FJ, Persson KE, Mugyenyi CK, Fowkes FJ, Simpson JA (2008). Acquisition of growth-inhibitory antibodies against blood-stage *Plasmodium falciparum*.. PLoS One.

[7] Bouharoun-Tayoun H, Attanath P, Sabchareon A, Chongsuphajaisiddhi T, Druilhe P (1990). Antibodies that protect humans against *Plasmodium falciparum* blood stages do not on their own inhibit parasite growth and invasion in vitro, but act in cooperation with monocytes.. J Exp Med.

[8] Hodder AN, Crewther PE, Anders RF (2001). Specificity of the protective antibody response to apical membrane antigen 1.. Infect Immun.

[9] Gaur D, Mayer DC, Miller LH (2004). Parasite ligand-host receptor interactions during invasion of erythrocytes by *Plasmodium* merozoites.. Int J Parasitol.

[10] O'Donnell RA, de Koning-Ward TF, Burt RA, Bockarie M, Reeder JC (2001). Antibodies against merozoite surface protein (MSP)-1(19) are a major component of the invasion-inhibitory response in individuals immune to malaria.. J Exp Med.

[11] Clark JT, Donachie S, Anand R, Wilson CF, Heidrich HG (1989). 46–53 kilodalton glycoprotein from the surface of *Plasmodium falciparum* merozoites.. Mol Biochem Parasitol.

[12] Oeuvray C, Bouharoun-Tayoun H, Gras-Masse H, Bottius E, Kaidoh T (1994). Merozoite surface protein-3: a malaria protein inducing antibodies that promote *Plasmodium falciparum* killing by cooperation with blood monocytes.. Blood.

[13] Theisen M, Soe S, Oeuvray C, Thomas AW, Vuust J (1998). The glutamate-rich protein (GLURP) of *Plasmodium falciparum* is a target for antibody-dependent monocyte-mediated inhibition of parasite growth in vitro.. Infect Immun.

[14] Dutta S, Haynes JD, Moch JK, Barbosa A, Lanar DE (2003). Invasion-inhibitory antibodies inhibit proteolytic processing of apical membrane antigen 1 of *Plasmodium falciparum* merozoites.. Proc Natl Acad Sci USA.

[15] Persson KE, McCallum FJ, Reiling L, Lister NA, Stubbs J (2008). Variation in use of erythrocyte invasion pathways by *Plasmodium falciparum* mediates evasion of human inhibitory antibodies.. J Clin Invest.

[16] Cowman AF, Crabb BS (2006). Invasion of red blood cells by malaria parasites.. Cell.

[17] Richards JS, Beeson JG (2009). The future for blood-stage vaccines against malaria.. Immunol Cell Biol.

[18] Gupta S, Snow RW, Donnelly CA, Marsh K, Newbold C (1999). Immunity to non-cerebral severe malaria is acquired after one or two infections.. Nat Med.

[19] Stroup DF, Berlin JA, Morton SC, Olkin I, Williamson GD (2000). Meta-analysis of observational studies in epidemiology: a proposal for reporting. Meta-analysis Of Observational Studies in Epidemiology (MOOSE) group.. JAMA.

[20] World Health Organization (1990). Severe and complicated malaria. World Health Organization, Division of Control of Tropical Diseases.. Trans R Soc Trop Med Hyg.

[21] World Health Organization (2000). Severe falciparum malaria. World Health Organization, Communicable Diseases Cluster.. Trans R Soc Trop Med Hyg.

[22] Egger M, Smith GD, Phillips AN (1997). Meta-analysis: principles and procedures.. BMJ.

[23] Zhang J, Yu KF (1998). What's the relative risk? A method of correcting the odds ratio in cohort studies of common outcomes.. JAMA.

[24] Higgins JP, Thompson SG, Deeks JJ, Altman DG (2003). Measuring inconsistency in meta-analyses.. BMJ.

[25] Ioannidis JP, Patsopoulos NA, Evangelou E (2007). Uncertainty in heterogeneity estimates in meta-analyses.. BMJ.

[26] Sterne JA, Egger M (2001). Funnel plots for detecting bias in meta-analysis: guidelines on choice of axis.. J Clin Epidemiol.

[27] Egger M, Davey Smith G, Schneider M, Minder C (1997). Bias in meta-analysis detected by a simple, graphical test.. BMJ.

[28] Meraldi V, Nebie I, Tiono AB, Diallo D, Sanogo E (2004). Natural antibody response to *Plasmodium falciparum* Exp-1, MSP-3 and GLURP long synthetic peptides and association with protection.. Parasite Immunol.

[29] Nebie I, Diarra A, Ouedraogo A, Soulama I, Bougouma EC (2008). Humoral responses to *Plasmodium falciparum* blood-stage antigens and association with incidence of clinical malaria in children living in an area of seasonal malaria transmission in Burkina Faso, West Africa.. Infect Immun.

[30] Nebie I, Tiono AB, Diallo DA, Samandoulougou S, Diarra A (2008). Do antibody responses to malaria vaccine candidates influenced by the level of malaria transmission protect from malaria?. Trop Med Int Health.

[31] Conway DJ, Cavanagh DR, Tanabe K, Roper C, Mikes ZS (2000). A principal target of human immunity to malaria identified by molecular population genetic and immunological analyses.. Nat Med.

[32] Polley SD, Tetteh KK, Cavanagh DR, Pearce RJ, Lloyd JM (2003). Repeat sequences in block 2 of *Plasmodium falciparum* merozoite surface protein 1 are targets of antibodies associated with protection from malaria.. Infect Immun.

[33] Metzger WG, Okenu DM, Cavanagh DR, Robinson JV, Bojang KA (2003). Serum IgG3 to the *Plasmodium falciparum* merozoite surface protein 2 is strongly associated with a reduced prospective risk of malaria.. Parasite Immunol.

[34] Polley SD, Tetteh KK, Lloyd JM, Akpogheneta OJ, Greenwood BM (2007). *Plasmodium falciparum* merozoite surface protein 3 is a target of allele-specific immunity and alleles are maintained by natural selection.. J Infect Dis.

[35] Dziegiel M, Rowe P, Bennett S, Allen SJ, Olerup O (1993). Immunoglobulin M and G antibody responses to *Plasmodium falciparum* glutamate-rich protein: correlation with clinical immunity in Gambian children.. Infect Immun.

[36] Egan AF, Morris J, Barnish G, Allen S, Greenwood BM (1996). Clinical immunity to *Plasmodium falciparum* malaria is associated with serum antibodies to the 19-kDa C-terminal fragment of the merozoite surface antigen, PfMSP-1.. J Infect Dis.

[37] Taylor RR, Allen SJ, Greenwood BM, Riley EM (1998). IgG3 antibodies to *Plasmodium falciparum* merozoite surface protein 2 (MSP2): increasing prevalence with age and association with clinical immunity to malaria.. Am J Trop Med Hyg.

[38] Okenu DM, Riley EM, Bickle QD, Agomo PU, Barbosa A (2000). Analysis of human antibodies to erythrocyte binding antigen 175 of *Plasmodium falciparum*.. Infect Immun.

[39] Okech BA, Corran PH, Todd J, Joynson-Hicks A, Uthaipibull C (2004). Fine specificity of serum antibodies to *Plasmodium falciparum* merozoite surface protein, PfMSP-1(19), predicts protection from malaria infection and high-density parasitemia.. Infect Immun.

[40] Gray JC, Corran PH, Mangia E, Gaunt MW, Li Q (2007). Profiling the antibody immune response against blood stage malaria vaccine candidates.. Clin Chem.

[41] Dodoo D, Theander TG, Kurtzhals JA, Koram K, Riley E (1999). Levels of antibody to conserved parts of *Plasmodium falciparum* merozoite surface protein 1 in Ghanaian children are not associated with protection from clinical malaria.. Infect Immun.

[42] Dodoo D, Theisen M, Kurtzhals JA, Akanmori BD, Koram KA (2000). Naturally acquired antibodies to the glutamate-rich protein are associated with protection against *Plasmodium falciparum* malaria.. J Infect Dis.

[43] Cavanagh DR, Dodoo D, Hviid L, Kurtzhals JA, Theander TG (2004). Antibodies to the N-terminal block 2 of *Plasmodium falciparum* merozoite surface protein 1 are associated with protection against clinical malaria.. Infect Immun.

[44] Dodoo D, Aikins A, Asamoah Kusi K, Lamptey H, Remarque E (2008). Cohort study of the association of antibody levels to AMA1, MSP119, MSP3 and GLURP with protection from clinical malaria in Ghanaian children.. Malar J.

[45] Polley SD, Mwangi T, Kocken CH, Thomas AW, Dutta S (2004). Human antibodies to recombinant protein constructs of *Plasmodium falciparum* Apical Membrane Antigen 1 (AMA1) and their associations with protection from malaria.. Vaccine.

[46] Polley SD, Conway DJ, Cavanagh DR, McBride JS, Lowe BS (2006). High levels of serum antibodies to merozoite surface protein 2 of *Plasmodium falciparum* are associated with reduced risk of clinical malaria in coastal Kenya.. Vaccine.

[47] Osier FH, Polley SD, Mwangi T, Lowe B, Conway DJ (2007). Naturally acquired antibodies to polymorphic and conserved epitopes of *Plasmodium falciparum* merozoite surface protein 3.. Parasite Immunol.

[48] Al-Yaman F, Genton B, Anders R, Taraika J, Ginny M (1995). Assessment of the role of the humoral response to *Plasmodium falciparum* MSP2 compared to RESA and SPf66 in protecting Papua New Guinean children from clinical malaria.. Parasite Immunol.

[49] Al-Yaman F, Genton B, Kramer K, Chang S, Hui G (1996). Assessment of the role of naturally acquired antibody levels to *Plasmodium falciparum* merozoite surface protein-1 in protecting Papa New Guinean children from malaria morbidity.. Am J Trop Med Hyg.

[50] Perraut R, Marrama L, Diouf B, Sokhna C, Tall A (2005). Antibodies to the conserved C-terminal domain of the *Plasmodium falciparum* merozoite surface protein 1 and to the merozoite extract and their relationship with in vitro inhibitory antibodies and protection against clinical malaria in a Senegalese village.. J Infect Dis.

[51] Lusingu JP, Vestergaard LS, Alifrangis M, Mmbando BP, Theisen M (2005). Cytophilic antibodies to *Plasmodium falciparum* glutamate rich protein are associated with malaria protection in an area of holoendemic transmission.. Malar J.

[52] John CC, O'Donnell RA, Sumba PO, Moormann AM, de Koning-Ward TF (2004). Evidence that invasion-inhibitory antibodies specific for the 19-kDa fragment of merozoite surface protein-1 (MSP-1 19) can play a protective role against blood-stage *Plasmodium falciparum* infection in individuals in a malaria endemic area of Africa.. J Immunol.

[53] John CC, Moormann AM, Pregibon DC, Sumba PO, McHugh MM (2005). Correlation of high levels of antibodies to multiple pre-erythrocytic *Plasmodium falciparum* antigens and protection from infection.. Am J Trop Med Hyg.

[54] Tolle R, Fruh K, Doumbo O, Koita O, N'Diaye M (1993). A prospective study of the association between the human humoral immune response to *Plasmodium falciparum* blood stage antigen gp190 and control of malarial infections.. Infect Immun.

[55] Stanisic DI, Richards JS, McCallum FJ, Michon P, King CL (2009). IgG subclass-specific responses against *Plasmodium falciparum* merozoite antigens are associated with control of parasitemia and protection from symptomatic illness.. Infect Immun.

[56] Perraut R, Marrama L, Diouf B, Fontenille D, Tall A (2003). Distinct surrogate markers for protection against *Plasmodium falciparum* infection and clinical malaria identified in a Senegalese community after radical drug cure.. J Infect Dis.

[57] Wang L, Richie TL, Stowers A, Nhan DH, Coppel RL (2001). Naturally acquired antibody responses to *Plasmodium falciparum* merozoite surface protein 4 in a population living in an area of endemicity in Vietnam.. Infect Immun.

[58] Scopel KK, da Silva-Nunes M, Malafronte RS, Braga EM, Ferreira MU (2007). Variant-specific antibodies to merozoite surface protein 2 and clinical expression of *Plasmodium falciparum* malaria in rural Amazonians.. Am J Trop Med Hyg.

[59] Osier FH, Fegan G, Polley SD, Murungi L, Verra F (2008). Breadth and magnitude of antibody responses to multiple *Plasmodium falciparum* merozoite antigens are associated with protection from clinical malaria.. Infect Immun.

[60] Sarr JB, Pelleau S, Toly C, Guitard J, Konate L (2006). Impact of red blood cell polymorphisms on the antibody response to *Plasmodium falciparum* in Senegal.. Microbes Infect.

[61] Koussis K, Withers-Martinez C, Yeoh S, Child M, Hackett F (2009). A multifunctional serine protease primes the malaria parasite for red blood cell invasion.. Embo J.

[62] Huber W, Felger I, Matile H, Lipps HJ, Steiger S (1997). Limited sequence polymorphism in the *Plasmodium falciparum* merozoite surface protein 3.. Mol Biochem Parasitol.

[63] Hu J, Chen Z, Gu J, Wan M, Shen Q (2008). Safety and immunogenicity of a malaria vaccine, *Plasmodium falciparum* AMA-1/MSP-1 chimeric protein formulated in montanide ISA 720 in healthy adults.. PLoS One.

[64] Malkin E, Hu J, Li Z, Chen Z, Bi X (2008). A phase 1 trial of PfCP2.9: an AMA1/MSP1 chimeric recombinant protein vaccine for *Plasmodium falciparum* malaria.. Vaccine.

[65] Ogutu BR, Apollo OJ, McKinney D, Okoth W, Siangla J (2009). Blood stage malaria vaccine eliciting high antigen-specific antibody concentrations confers no protection to young children in Western Kenya.. PLoS ONE.

[66] Moher D, Fortin P, Jadad AR, Juni P, Klassen T (1996). Completeness of reporting of trials published in languages other than English: implications for conduct and reporting of systematic reviews.. Lancet.

[67] Roussilhon C, Oeuvray C, Muller-Graf C, Tall A, Rogier C (2007). Long-term clinical protection from falciparum malaria is strongly associated with IgG3 antibodies to merozoite surface protein 3.. PLoS Med.

[68] Mabunda S, Aponte JJ, Tiago A, Alonso P (2009). A country-wide malaria survey in Mozambique. II. Malaria attributable proportion of fever and establishment of malaria case definition in children across different epidemiological settings.. Malar J.

[69] Mwangi TW, Ross A, Snow RW, Marsh K (2005). Case definitions of clinical malaria under different transmission conditions in Kilifi District, Kenya.. J Infect Dis.

[70] Smith T, Schellenberg JA, Hayes R (1994). Attributable fraction estimates and case definitions for malaria in endemic areas.. Stat Med.

[71] Bejon P, Warimwe G, Mackintosh CL, Mackinnon MJ, Kinyanjui SM (2009). Analysis of immunity to febrile malaria in children that distinguishes immunity from lack of exposure.. Infect Immun.

[72] Egan AF, Burghaus P, Druilhe P, Holder AA, Riley EM (1999). Human antibodies to the 19kDa C-terminal fragment of *Plasmodium falciparum* merozoite surface protein 1 inhibit parasite growth in vitro.. Parasite Immunol.

[73] Vandenbroucke JP, von Elm E, Altman DG, Gotzsche PC, Mulrow CD (2007). Strengthening the Reporting of Observational Studies in Epidemiology (STROBE): explanation and elaboration.. Ann Intern Med.

[74] von Elm E, Altman DG, Egger M, Pocock SJ, Gotzsche PC (2007). The Strengthening the Reporting of Observational Studies in Epidemiology (STROBE) statement: guidelines for reporting observational studies.. Lancet.

