# Supplementary material for: The Relationship between Anti-merozoite Antibodies and Incidence of Plasmodium falciparum Malaria: A Systematic Review and Meta-analysis
Source: PLoS Med. 2010 Jan 19;7(1):e1000218. doi: 10.1371/journal.pmed.1000218 (PMC2808214; doi:10.1371/journal.pmed.1000218)
Supplement: Text S2 — Excluded studies, supplementary tables, and analyses. (0.54 MB DOC) [file pmed.1000218.s002.doc]

**Text S2. Supporting information**

**The Relationship between Anti-merozoite Antibodies and Incidence of *Plasmodium falciparum* Malaria: A Systematic Review and Meta-Analysis**

Freya J.I. Fowkes, Jack S. Richards, Julie A. Simpson and James G. Beeson

[**Supporting information I:** Details of excluded studies 2](#__RefHeading___Toc244153544)

[**Supporting information II:** Supplementary Tables 10](#__RefHeading___Toc244153546)

[Table A. Details of MSP-1 recombinant antigens featured in this systematic review 10](#__RefHeading___Toc244153547)

[Table B. Details of other recombinant antigens featured in this systematic review 12](#__RefHeading___Toc244153548)

[**Supporting information III:** Supplementary Analyses - Association between anti-merozoite antibodies and incidence of *P. falciparum* reinfection and high density *P. falciparum* 14](#__RefHeading___Toc244153549)

[MSP-1 14](#__RefHeading___Toc244153550)

[MSP-2 15](#__RefHeading___Toc244153551)

[AMA-1 15](#__RefHeading___Toc244153552)

[**Supporting information IV:** MSP-1-EGF individual study estimates 16](#__RefHeading___Toc244153553)

[MSP-1-EGF1 16](#__RefHeading___Toc244153554)

[MSP-1-EGF2 16](#__RefHeading___Toc244153555)

# Supporting information I: Details of excluded studies

**
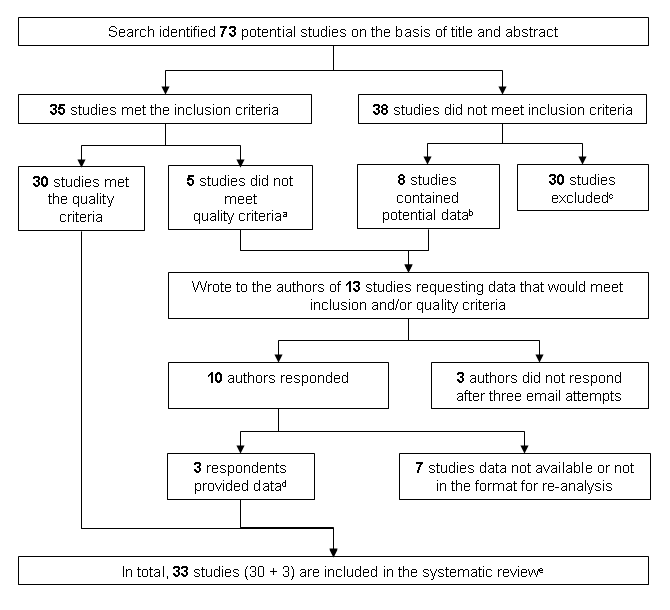
**

Figure 1 in manuscript.

Databases were searched and the title and abstract of search results were examined. We identified 73 potential studies on the basis of information provided in the title and abstract. In many cases, the study design and/or study population had been inadequately described in the title and/or abstract so a more detailed review of these papers was warranted. After reading all 71 papers, we found that a large number of studies (n = 38) did not meet the inclusion criteria (Figure 1).

INCLUSION CRITERIA

Details of inclusion and exclusion criteria can be found on pages 7-9 of the manuscript. Briefly, the criteria for inclusion of studies were population-based prospective studies and population-based treatment to reinfection studies, with antibodies measured at baseline. The criterion for inclusion of participants was individuals living in malaria endemic areas.

In total 30 studies were excluded outright (Figure 1):

- 15 studies analyzed antibody and malaria data collected from cross-sectional studies (including serial cross-sectional studies) [1-15]. These studies were excluded because a temporal relationship between antibodies and malaria cannot be established from data collected at the same time point
- 6 were studies that recruited individuals based on their clinical status [16-21] and were excluded because participants would not be representative of the general population
- 3 were mother/infant studies [22-24] and were excluded to remove the confounding effect of placental-transferred maternal antibodies
- 3 studies were excluded because they were sero-prevalence surveys and did not relate antibody response to *P. falciparum* malaria outcome [25-27]
- 2 studies were excluded because outcome was clinical failure of anti-malarial treatment [28,29]
- 1 study measured IgG responses to undefined regions of antigens [30].

Of the 37 studies that did not meet inclusion criteria, we also identified 8 studies that contained data [31-38] and could potentially be reanalyzed to meet our inclusion criteria:

- 3 studies related antibody data to both retrospectively and prospectively collected data [31-33]
- 1 study had restricted analysis to those who were parasite positive but had collected data on whole study cohort [34]
- 4 studies had analyzed data as differences in antibody levels at baseline in those who subsequently developed *P. falciparum* malaria and those who did not [35-38]

In addition, we identified 5 studies that met inclusion criteria but not quality criteria regarding the definition of symptomatic malaria and could potentially be reanalyzed to meet our quality criteria (Figure 1). Definitions included:

- fever/history of fever plus a *P. falciparum* parasitaemia of any density [39-41],
- nine common symptoms, but fever definition not reached in all cases [42]
- *P. falciparum* positive plus fever and clinical symptoms and in absence of symptoms, >5000/ul for children and >2000/ul for adults [43]

To maximize the number of included studies, we wrote to the corresponding author of the 13 studies that could potentially be reanalyzed [31-43]. Three corresponding authors provided data for inclusion in the meta-analysis [34,36,42]. The remainder either responded saying that the data was unavailable, or that it was not in a format for reanalysis, or did not respond to three separate emails.

**References for Supporting Information I:**

1. al-Yaman F, Genton B, Anders RF, Falk M, Triglia T, *et al*. (1994) Relationship between humoral response to *Plasmodium falciparum* merozoite surface antigen-2 and malaria morbidity in a highly endemic area of Papua New Guinea. Am J Trop Med Hyg 51: 593-602.

2. al-Yaman F, Genton B, Kramer KJ, Taraika J, Chang SP, *et al*. (1995) Acquired antibody levels to *Plasmodium falciparum* merozoite surface antigen 1 in residents of a highly endemic area of Papua New Guinea. Trans R Soc Trop Med Hyg 89: 555-559.

3. Braga EM, Barros RM, Reis TA, Fontes CJ, Morais CG, *et al*. (2002) Association of the IgG response to *Plasmodium falciparum* merozoite protein (C-terminal 19 kD) with clinical immunity to malaria in the Brazilian Amazon region. Am J Trop Med Hyg 66: 461-466.

4. Ford L, Lobo CA, Rodriguez M, Zalis MG, Machado RL, *et al*. (2007) Differential antibody responses to *Plasmodium falciparum* invasion ligand proteins in individuals living in malaria-endemic areas in Brazil and Cameroon. Am J Trop Med Hyg 77: 977-983.

5. Fruh K, Doumbo O, Muller H, Koita O, McBride J, *et al*. (1991) Human antibody response to the major merozoite surface antigen of *Plasmodium falciparum* is strain specific and short-lived. Infect Immun 59: 1319-1324.

6. Hogh B, Petersen E, Dziegiel M, David K, Hanson A, *et al*. (1992) Antibodies to a recombinant glutamate-rich *Plasmodium falciparum* protein: evidence for protection of individuals living in a holoendemic area of Liberia. Am J Trop Med Hyg 46: 307-313.

7. Ladeia-Andrade S, Ferreira MU, Scopel KK, Braga EM, Bastos Mda S, *et al*. (2007) Naturally acquired antibodies to merozoite surface protein (MSP)-1(19) and cumulative exposure to *Plasmodium falciparum* and *Plasmodium vivax* in remote populations of the Amazon Basin of Brazil. Mem Inst Oswaldo Cruz 102: 943-951.

8. Nebie I, Cuzin-Ouattara N, Diallo DA, Cousens SN, Theisen M, *et al*. (2003) Humoral responses to defined malaria antigens in children living since birth under insecticide treated curtains in Burkina Faso. Acta Trop 88: 17-25.

9. Okech BA, Nalunkuma A, Okello D, Pang XL, Suzue K, *et al*. (2001) Natural human immunoglobulin G subclass responses to *Plasmodium falciparum* serine repeat antigen in Uganda. Am J Trop Med Hyg 65: 912-917.

10. Riley E, Morris-Jones S, Blackman M, Greenwood B, Holder A (1993) A Longitudinal study of naturally acquired cellular and humoral immune responses to a merozoite surface protein (MSP1) of *Plasmodium falciparum* in an area of seasonal malaria transmission. Parasite Immunol 15: 513-524.

11. Scopel KK, Fontes CJ, Ferreira MU, Braga EM (2006) Factors associated with immunoglobulin G subclass polarization in naturally acquired antibodies to *Plasmodium falciparum* merozoite surface proteins: a cross-sectional survey in Brazilian Amazonia. Clin Vaccine Immunol 13: 810-813.

12. Thomas AW, Trape JF, Rogier C, Goncalves A, Rosario VE, *et al*. (1994) High prevalence of natural antibodies against *Plasmodium falciparum* 83-kilodalton apical membrane antigen (PF83/AMA-1) as detected by capture-enzyme-linked immunosorbent assay using full-length baculovirus recombinant PF83/AMA-1. Am J Trop Med Hyg 51: 730-740.

13. Torres KJ, Clark EH, Hernandez JN, Soto-Cornejo KE, Gamboa D, *et al*. (2008) Antibody response dynamics to the *Plasmodium falciparum* conserved vaccine candidate antigen, merozoite surface protein-1 C-terminal 19kD, in Peruvians exposed to hypoendemic malaria transmission. Malar J 7: 173.

14. Udhayakumar V, Kariuki S, Kolczack M, Girma M, Roberts JM, *et al*. (2001) Longitudinal study of natural immune responses to the *Plasmodium falciparum* apical membrane antigen (AMA-1) in a holoendemic region of malaria in western Kenya: Asembo Bay Cohort Project VIII. Am J Trop Med Hyg 65: 100-107.

15. Biswas S, Seth RK, Tyagi PK, Sharma SK, Dash AP (2008) Naturally acquired immunity and reduced susceptibility to falciparum malaria in two subpopulations of endemic eastern India. Scand J Immunol 67: 177-184.

16. Ekala MT, Jouin H, Lekoulou F, Mercereau-Puijalon O, Ntoumi F (2002) Allelic family-specific humoral responses to merozoite surface protein 2 (MSP2) in Gabonese residents with *Plasmodium falciparum* infections. Clin Exp Immunol 129: 326-331.

17. Kinyanjui SM, Conway DJ, Lanar DE, Marsh K (2007) IgG antibody responses to *Plasmodium falciparum* merozoite antigens in Kenyan children have a short half-life. Malar J 6: 82.

18. Kohler C, Tebo AE, Dubois B, Deloron P, Kremsner PG, *et al*. (2003) Temporal variations in immune responses to conserved regions of *Plasmodium falciparum* merozoite surface proteins related to the severity of a prior malaria episode in Gabonese children. Trans R Soc Trop Med Hyg 97: 455-461.

19. Iriemenam NC, Khirelsied AH, Nasr A, Elghazali G, Giha HA, *et al*. (2008) Antibody responses to a panel of *Plasmodium falciparum* malaria blood-stage antigens in relation to clinical disease outcome in Sudan. Vaccine 27: 62-71.

20. Woodberry T, Minigo G, Piera KA, Hanley JC, de Silva HD, *et al*. (2008) Antibodies to *Plasmodium falciparum* and *Plasmodium vivax* merozoite surface protein 5 in Indonesia: species-specific and cross-reactive responses. J Infect Dis 198: 134-142.

21. Migot-Nabias F, Luty AJ, Ringwald P, Vaillant M, Dubois B, *et al*. (1999) Immune responses against *Plasmodium falciparum* asexual blood-stage antigens and disease susceptibility in Gabonese and Cameroonian children. Am J Trop Med Hyg 61: 488-494.

22. Dent A, Malhotra I, Mungai P, Muchiri E, Crabb BS, *et al*. (2006) Prenatal malaria immune experience affects acquisition of *Plasmodium falciparum* merozoite surface protein-1 invasion inhibitory antibodies during infancy. J Immunol 177: 7139-7145.

23. Kitua AY, Urassa H, Wechsler M, Smith T, Vounatsou P, *et al*. (1999) Antibodies against *Plasmodium falciparum* vaccine candidates in infants in an area of intense and perennial transmission: relationships with clinical malaria and with entomological inoculation rates. Parasite Immunol 21: 307-317.

24. Riley EM, Wagner GE, Ofori MF, Wheeler JG, Akanmori BD, *et al*. (2000) Lack of association between maternal antibody and protection of African infants from malaria infection. Infect Immun 68: 5856-5863.

25. Jouin H, Rogier C, Trape JF, Mercereau-Puijalon O (2001) Fixed, epitope-specific, cytophilic antibody response to the polymorphic block 2 domain of the *Plasmodium falciparum* merozoite surface antigen MSP-1 in humans living in a malaria-endemic area. Eur J Immunol 31: 539-550.

26. Noland GS, Hendel-Paterson B, Min XM, Moormann AM, Vulule JM, *et al*. (2008) Low prevalence of antibodies to pre-erythrocytic but not blood-stage *Plasmodium falciparum* antigens in an area of unstable as compared to stable malaria transmission. Infect Immun 76: 5721-5728.

27. Akpogheneta OJ, Duah NO, Tetteh KK, Dunyo S, Lanar DE, *et al*. (2008) Duration of naturally acquired antibody responses to blood-stage *Plasmodium falciparum* is age dependent and antigen specific. Infect Immun 76: 1748-1755.

28. Aubouy A, Migot-Nabias F, Deloron P (2007) Correlations between treatment outcome and both anti-MSP119 antibody response and erythrocyte-related genetic factors in *Plasmodium falciparum* malaria. Infect Genet Evol 7: 147-154.

29. Pinder M, Sutherland CJ, Sisay-Joof F, Ismaili J, McCall MB, *et al*. (2006) Immunoglobulin G antibodies to merozoite surface antigens are associated with recovery from chloroquine-resistant *Plasmodium falciparum* in Gambian children. Infect Immun 74: 2887-2893.

30. Topolska AE, Richie TL, Nhan DH, Coppel RL (2004) Associations between responses to the rhoptry-associated membrane antigen of *Plasmodium falciparum* and immunity to malaria infection. Infect Immun 72: 3325-3330.

31. Oeuvray C, Theisen M, Rogier C, Trape JF, Jepsen S, *et al*. (2000) Cytophilic immunoglobulin responses to *Plasmodium falciparum* glutamate-rich protein are correlated with protection against clinical malaria in Dielmo, Senegal. Infect Immun 68: 2617-2620.

32. Roussilhon C, Oeuvray C, Muller-Graf C, Tall A, Rogier C, *et al*. (2007) Long-term clinical protection from falciparum malaria is strongly associated with IgG3 antibodies to merozoite surface protein 3. PLoS Med 4: e320.

33. Soe S, Theisen M, Roussilhon C, Aye KS, Druilhe P (2004) Association between protection against clinical malaria and antibodies to merozoite surface antigens in an area of hyperendemicity in Myanmar: complementarity between responses to merozoite surface protein 3 and the 220-kilodalton glutamate-rich protein. Infect Immun 72: 247-252.

34. Osier FH, Fegan G, Polley SD, Murungi L, Verra F, *et al*. (2008) Breadth and magnitude of antibody responses to multiple *Plasmodium falciparum* merozoite antigens are associated with protection from clinical malaria. Infect Immun 76: 2240-2248.

35. Riley E, Allen S, Wheeler J, Blackman M, Bennet S, *et al*. (1992) Naturally acquired cellular and humoral immune responses to the major merozoite surface antigen (PfMSP1) of *Plasmodium falciparum* are associated with reduced malaria morbidity. Parasite Immunol 14: 321-337.

36. Sarr JB, Pelleau S, Toly C, Guitard J, Konate L, *et al*. (2006) Impact of red blood cell polymorphisms on the antibody response to *Plasmodium falciparum* in Senegal. Microbes Infect 8: 1260-1268.

37. Theisen M, Dodoo D, Toure-Balde A, Soe S, Corradin G, *et al*. (2001) Selection of glutamate-rich protein long synthetic peptides for vaccine development: antigenicity and relationship with clinical protection and immunogenicity. Infect Immun 69: 5223-5229.

38. Wang L, Crouch L, Richie TL, Nhan DH, Coppel RL (2003) Naturally acquired antibody responses to the components of the *Plasmodium falciparum* merozoite surface protein 1 complex. Parasite Immunol 25: 403-412.

39. Cavanagh DR, Elhassan IM, Roper C, Robinson VJ, Giha H, *et al*. (1998) A longitudinal study of type-specific antibody responses to *Plasmodium falciparum* merozoite surface protein-1 in an area of unstable malaria in Sudan. J Immunol 161: 347-359.

40. Fonjungo PN, Elhassan IM, Cavanagh DR, Theander TG, Hviid L, *et al*. (1999) A longitudinal study of human antibody responses to *Plasmodium falciparum* rhoptry-associated protein 1 in a region of seasonal and unstable malaria transmission. Infect Immun 67: 2975-2985.

41. Lusingu JP, Jensen AT, Vestergaard LS, Minja DT, Dalgaard MB, *et al*. (2006) Levels of plasma immunoglobulin G with specificity against the cysteine-rich interdomain regions of a semiconserved *Plasmodium falciparum* erythrocyte membrane protein 1, VAR4, predict protection against malarial anemia and febrile episodes. Infect Immun 74: 2867-2875.

42. Scopel KK, da Silva-Nunes M, Malafronte RS, Braga EM, Ferreira MU (2007) Variant-specific antibodies to merozoite surface protein 2 and clinical expression of *Plasmodium falciparum* malaria in rural Amazonians. Am J Trop Med Hyg 76: 1084-1091.

43. Aucan C, Traore Y, Tall F, Nacro B, Traore-Leroux T, *et al*. (2000) High immunoglobulin G2 (IgG2) and low IgG4 levels are associated with human resistance to *Plasmodium falciparum* malaria. Infect Immun 68: 1252-1258.

# Supporting information II: Supplementary Tables

## Table A. Details of MSP-1 recombinant antigens featured in this systematic review

| **Author, year [reference]** | **Allele/ Strain (region)** | **Expression System** | **Tag** | **Sero-prevalence** |
| --- | --- | --- | --- | --- |
| **MSP-1_19_** |  |  |  |  |
| Cavanagh, 2004[43] | Wellcome (1631-1726) | *E. coli* | GST | 56% |
| Conway, 2000[31] | Wellcome (1631-1726) | *E. coli* | GST | 63% |
| Dodoo, 2008[44] | Palo Alto (1588-1760) | Baculovirus | His | DNS |
| Egan, 1996[36] | MAD20 (1631-1744) | *S. cerevisiae* | His | 17%(SL), 9%(G) |
| John, 2004[53] | MAD20 (1726-1744) | *S. cerevisiae* | His | 50% |
| Nebie, 2008[29] | Uganda Palo Alto (20-43, 1615-1723) | Baculovirus | His | DNS |
| Okech, 2004[39] | Wellcome (1631-1744) | *E. coli* | GST | 60% |
| Osier, 2008[59] | Wellcome (1631-1726) | *E. coli* | GST | 48% |
| Perraut, 2003[56] | Palo Alto (DNS) | Baculovirus | GST | 79% |
| Perraut, 2005[50] | Palo Alto (DNS) | Baculovirus | GST | 77% |
| Stanisic, 2009[55] | 3D7 (1631-1744) | *E. coli* | His | 97% |
| Wang, 2001[57] | Wellcome (1631-1726) | *E. coli* | GST | 96% |
| **MSP-1-EGF1 and EGF2** | |  |  |  |
| Dodoo, 1999[41] | MAD20 (1631-1678) and (1674-1726) | *E. coli* | GST | 16%, 4% |
| Egan, 1996[36] | MAD20 (1631-1678) and (1674-1726) | *E. coli* | GST | 13%, 3% (SL), 6%, 3% (G) |
| **MSP-1_42_** |  |  |  |  |
| Al-Yaman, 1996[49] | Ugandan Palo Alto (1333-1705) | Baculovirus | DNS | DNS |
| **MSP-1-block 1** |  |  |  |  |
| Cavanagh, 2004[43] | MAD20 (21-53) | *E. coli* | GST | 1.4% |
| Conway, 2000[31] | MAD20 (21-53) | *E. coli* | GST | 3% |
| **MSP-1-block 2 (Full length)** | |  |  |  |
| Cavanagh, 2004[43] | K1 (54-144), MAD20 (54-112), RO33 (54-106) | *E. coli* | GST | 14%, 20%, 6% |
| Conway, 2000[31] | 3D7 (54-144), MAD20 (54-144), RO33 (54-144) | *E. coli* | GST | 22%, 24%, 15% |
| Gray, 2007[40] | 3D7 (54-144), MAD20 (54-144), RO33 (54-144) | *E. coli* | GST | 17%, 19%, 17% |
| Osier, 2008[59] | 3D7 (54-144), MAD20 (54-144), RO33 (54-144) | *E. coli* | GST | 24%, 16%, 24% |
| Tolle, 1993[54] | RO33 (56-105) | *E. coli* | His | DNS |
| **MSP-1-block 2 (flanks)** | |  |  |  |
| Cavanagh, 2004[43] | K1 (54-63, 120-144), MAD20 (54-71, 99-112) | *E. coli* | GST | 8%, 3% |
| Gray, 2007[40] | K1 (54-63, 120-144), MAD20 (54-71, 99-112) | *E. coli* | GST | 17%, 17% |
| Polley, 2003[32] | K1 (54-63, 120-144), MAD20 (54-71, 99-112) | *E. coli* | GST | 10%, 22% |
| **MSP-1-block 2 (repeats)** | |  |  |  |
| Cavanagh, 2004[43] | 3D7 (64-96), MAD20 (72-98) | *E. coli* | GST | 13%, 8% |
| Gray, 2007[40] | 3D7 (64-96), MAD20 (72-98) | *E. coli* | GST | 14%, 17% |
| Polley, 2003[32] | 3D7 (64-96), MAD20 (72-98) | *E. coli* | GST | 10%, 14% |

Region shows amino acid positions. If no position is given then BLAST searches were performed with amino acid sequence. Abbreviations: Ct, C-terminal; DNS, data not shown in original manuscript; G, The Gambia; GST, Glutathione *S*-Transferase; His, histadine; MSP, Maltose-Binding Protein; NA, Not Applicable; SL, Sierra-Leone. Please see main manuscript for references.

## Table B. Details of other recombinant antigens featured in this systematic review

| **Author, year**  **[reference]** | **Allele/ Strain (region)** | **Expression System** | **Tag** | **Sero-prevalence** |
| --- | --- | --- | --- | --- |
| **MSP-2** |  |  |  |  |
| Al-Yaman, 1995[48] | 3D7, FC27 ("Near to full length") | *E. coli* | His | DNS |
| Metzger, 2003[33] | 3D7-like [T9/96] (22-286), FC27-like [Dd2] (22-247), K1 (Ct-207-263) | *E. coli* | GST | 55%, 52%, 5.2% |
| Polley, 2006[46] | 3D7-like [CH150/9] (1-184), FC27-like [Dd2] (22-247) | *E. coli* | GST | 78%, 79% |
| Scopel*,* 2007[58] | 3D7 (44-104), FC27 (67-220) | *E. coli* | GST | 6%, 22% |
| Sarr, 2006[60] | 3D7 (19-249), FC27 (19-241) | *E. coli* | His | 81%, 95% |
| Stanisic, 2009[55] | 3D7 (1-272), FC27 (1-272) | *E. coli* | His | 86%, 83% |
| Taylor, 1998[37] | 3D7-like [T9/96] (22-286), FC27-like [Dd2] (22-247) | *E. coli* | GST | 18%, 13% |
| **MSP-3** |  |  |  |  |
| Dodoo, 2008[44] | FC27 (Ct-181-276) | SP | NA | DNS |
| Gray, 2007[40] | K1 (2-379), 3D7 (2-354) | *E. coli* | MBP | 25%, 15% |
| Meraldi, 2004[28] | 3D7 (Ct-154-249) | SP | NA | 57% |
| Nebie, 2008[30] | DNS (Ct-181-276) | DNS | DNS | DNS |
| Nebie, 2008[29] | FC27 (Ct-181-276) | SP | NA | DNS |
| Osier, 2007[47] | K1 (2-379), 3D7 (2-354), 3D7 (Ct-234-354) | *E. coli* | MBP | 41%, 54%, 24% |
| Polley, 2007[34] | K1 (2-379), 3D7 (2-354), 3D7 (Ct-234-354) | *E. coli* | MBP | 64%, 61%, 22% |
| **MSP-4 and MSP-4-EGF** | |  |  |  |
| Wang, 2001[57] | D10 (1-272) and D10 (204-248) | *E. coli* | His | 83% |
| **AMA-1** |  |  |  |  |
| Dodoo, 2008[44] | FVO (25-545) | *P. pastoris* | His | DNS |
| Gray, 2007[40] | 3D7 (85-531) | *E. coli* | His | 44% |
| John, 2005[52] | 3D7 (1-622) | *P. pastoris* | His | 88% |
| Nebie, 2008[29] | FVO (25-545) | *P. pastoris* | His | DNS |
| Polley, 2004[45] | FVO (25-544), 3D7 (85-531) | *P. pastoris, E. coli* | His | 67%, 70% |
| Stanisic, 2009[55] | 3D7 (85-531) | *E. coli* | His | 99.5% |
| **EBA-175** |  |  |  |  |
| John, 2005[53] | 3D7 (RII-145-760) | *P. pastoris* | None | 72% |
| Okenu, 2000 [38] | 3D7 (RII-144-753) | Baculovirus | His | 43% |
| Osier, 2008[59] | 3D7 (F2-461-753) | Baculovirus | GST | 74% |
| **GLURP** |  |  |  |  |
| Dziegiel, 1993[35] | DNS (R1-R2-489-1271) | *E. coli* | β-gala | 48% |
| Dodoo, 2000[42] | F32 (R0-94-489), F32 (R1-489-705), F32 (R2-705-1178) | *E. coli* | His | DNS |
| Dodoo, 2008[44] | F32 (R0-25-514) | *E. coli* | His | DNS |
| Lusingu, 2005[51] | DNS (R0-25-500) | DNS | His | DNS |
| Meraldi, 2004[28] | 3D7 (R2-801-920) | SP | NA | 68% |
| Nebie, 2008[29] | F32 (R0-25-514) | *E. coli* | His | DNS |
| Nebie, 2008 [30] | F32 (R0-94-489), F32 (R2-705-1178) | *E. coli* | His | DNS |

Region shows amino acid positions. If no position is given then BLAST searches were performed with amino acid sequence. Abbreviations: β-gala, β-galactosidase; Ct, C-terminal; DNS, data not shown in original manuscript; G, The Gambia; GST, Glutathione *S*-Transferase; His, histadine; MSP, Maltose-Binding Protein; NA, Not Applicable; SP, Synthetic peptide. Please see main manuscript for references.

# Supporting information III: Supplementary Analyses - Association between anti-merozoite antibodies and incidence of *P. falciparum* reinfection and high density *P. falciparum*

## MSP-1

Two studies examined the association of MSP-1_19_ antibodies with risk of high density *P. falciparum* infection. Stanisic *et al* (2009) showed a reduced risk of high density infection in those with high anti-MSP-1_19_ levels (>66%tile versus <33%tile RR 0.47, 95%CI 0.25, 0.87, *P* = 0.019; 33-66%tile versus <33%tile RR 0.7, 95%CI 0.41, 1.2, *P* = 0.19) whereas Okech *et al* (2004) showed no evidence of an association (responders versus non-responders, RR 0.88 95%CI 0.45, 1.73) [39,55].

Pooled results of two studies showed no evidence of an association between IgG responses to MSP-1_19_ and risk of reinfection (responder v non-responder RR 1.15, 95%CI 0.64, 2.07, *P* = 0.65) [52,57]. Stanisic *et al* (2009) also showed no association with reinfection (>66%tile versus <33%tile, RR 1.17, 95%CI 0.77, 1.78, *P* = 0.46; 33-66%tile versus <33%tile, RR = 1.22, 95%CI 0.83, 1.79, *P* = 0.31) [55]. Weak evidence of an association was seen when examining anti-MSP-1-9 levels [56] (transformation if any not stated) and risk of reinfection (RR 0.97, 95%CI 0.94, 1.00, *P* = 0.0928). One additional study showed that MSP-1-block 2 (RO33) responders had increased risk of reinfection compared to non-responders (RR 2.37, 95%CI 1.4, 3.99, *P* = 0.001) [54].

## MSP-2

Stanisic *et al* (2009) found no association between antibodies to MSP-2_FC27_ or MSP-2_3D7_ and risk of reinfection (responders versus non-responders RR 1.07, 95%CI 0.69, 1.64, *P* = 0.77 and RR 0.85, 95%CI 0.52, 1.37, *P* = 0.5 respectively) [55]. No association with risk of high density infection was seen with either antigen (MSP-2_FC27_, responder versus non-responder RR 0.59, 95%CI 0.36, 1.03, *P* = 0.065; MSP-2_3D7_, RR 0.66, 95%CI 0.35, 1.24, *P* = 0.2) [55].

## AMA-1

One study showed no association of AMA-1_3D7_ antibodies with risk of reinfection (>75%tile versus <75%tile, RR 0.74, 95%CI 0.41, 1.33, *P* = 0.32) whereas another showed increased risk of reinfection (>66%tile versus <33%tile, RR 1.64, 95%CI 1.11, 2.43, *P* = 0.013; 33-66%tile versus <33%tile, RR = 1.23, 95%CI 0.84, 1.82, *P* = 0.29) [53,55]. No evidence of an association of anti-AMA-1_3D7_ responses with risk of high density infection was found in another study (>66%tile versus <33%tile, RR 0.83, 95%CI 0.48, 1.44, *P* = 0.5; 33-66%tile versus <33%tile, RR = 0.76, 95%CI 0.44, 1.32, *P* = 0.33) [55].

# Supporting information IV: MSP-1-EGF individual study estimates

## MSP-1-EGF1

Pooled results of MSP-1-EGF1 studies showed no association between antibody responders with protection against symptomatic *P. falciparum* (RR 1.06, 95%CI 0.88, 1.26, *P* = 0.56, I^2^ = 0% 95%CI 0%, 87.3%). Individual study estimates were RR 1.19, 95%CI 0.86, 1.65 for Dodoo *et al* (1999)[41], 0.94, 95%CI 0.73, 1.21 and 1.19, 95%CI 0.78, 1.81 for Egan *et al* (1996)[36] in Sierra Leone and The Gambia respectively. All estimates are unadjusted and were calculated by the current authors by data provided in the paper.

## MSP-1-EGF2

Pooled results of MSP-1-EGF1 studies showed no association between antibody responders with protection against symptomatic *P. falciparum* reRR 0.59, 95%CI 0.19, 1.84, *P* = 0.37; I^2^ = 71.4%, 95%CI 2.8-91.6%. Individual study estimates were RR 1.31, 95%CI 0.73, 2.35 for Dodoo *et al* (1999) [41] and 0.46, 95%CI 0.19, 1.09 and 0.08, 95%CI 0.005, 1.19 for Egan *et al* (1996) [36] in Sierra Leone and The Gambia respectively. All estimates are unadjusted and were calculated by the current authors by data provided in the paper.
